# Reconstructing contact network parameters from viral phylogenies

**DOI:** 10.1093/ve/vew029

**Published:** 2016-10-30

**Authors:** Rosemary M. McCloskey, Richard H. Liang, Art F.Y. Poon

**Affiliations:** 1 BC Centre for Excellence in HIV/AIDS, Vancouver, Canada; 2 Department of Medicine, University of British Columbia, Vancouver, Canada

**Keywords:** phylogenetics, phylodynamics, contact network, transmission tree, approximate Bayesian computation, human immunodeficiency virus.

## Abstract

Models of the spread of disease in a population often make the simplifying assumption that the population is homogeneously mixed, or is divided into homogeneously mixed compartments. However, human populations have complex structures formed by social contacts, which can have a significant influence on the rate of epidemic spread. Contact network models capture this structure by explicitly representing each contact which could possibly lead to a transmission. We developed a method based on approximate Bayesian computation (ABC), a likelihood-free inference strategy, for estimating structural parameters of the contact network underlying an observed viral phylogeny. The method combines adaptive sequential Monte Carlo for ABC, Gillespie simulation for propagating epidemics though networks, and a kernel-based tree similarity score. We used the method to fit the Barabási-Albert network model to simulated transmission trees, and also applied it to viral phylogenies estimated from ten published HIV sequence datasets. This model incorporates a feature called preferential attachment (PA), whereby individuals with more existing contacts accumulate new contacts at a higher rate. On simulated data, we found that the strength of PA and the number of infected nodes in the network can often be accurately estimated. On the other hand, the mean degree of the network, as well as the total number of nodes, was not estimable with ABC. We observed sub-linear PA power in all datasets, as well as higher PA power in networks of injection drug users. These results underscore the importance of considering contact structures when performing phylodynamic inference. Our method offers the potential to quantitatively investigate the contact network structure underlying viral epidemics.

## Introduction

When an infectious disease spreads through a population, transmissions are generally more likely to occur between certain pairs of individuals. Such pairs must have a particular mode of contact with one another, which varies with the mode of transmission of the disease. For airborne pathogens, physical proximity may be sufficient, while for sexually transmitted diseases, sexual or in some cases blood-to-blood contact is required. The population together with the set of links between individuals along which transmission can occur is called the contact network (Klovdahl 1985; Morris 1993). The structure of the contact network underlying an epidemic can profoundly impact the speed and pattern of the epidemic’s expansion. Network structure can influence the prevalence curve (O’Dea and Wilke 2011; Ma, van den Driessche, and Willeboordse 2013) and transmission rate necessary for an epidemic to develop (Barthélemy et al. 2005). In turn, these estimates affect the estimates of quantities such as effective viral population size (Goodreau 2006). From a public health perspective, contact networks have been explored as tools for curtailing epidemic spread, by way of interventions targeted to well-connected nodes (Wang et al. 2015). True contact networks are a challenging type of data to collect, requiring extensive epidemiological investigation (Welch, Bansal, and Hunter, 2011; Eames et al. 2015 ).

Viral sequence data, on the other hand, has become easier to collect as the cost of sequencing has declined. In the case of HIV, genotyping has become part of routine clinical care in several health regions. Due to the high mutation rate of RNA viruses, epidemiological processes impact the course of viral evolution, thereby shaping the inter-host viral phylogeny (Drummond et al. 2003). The term ‘phylodynamics’ was coined to describe this interaction, as well as the growing family of inference methods to estimate epidemiological parameters from viral phylogenies (Grenfell et al. 2004). These methods have revealed diverse properties of local viral outbreaks, from basic reproductive number (Stadler et al. 2012), to the degree of clustering (Hughes et al. 2009), to the elevated transmission risk during acute infection (Volz et al. 2012). On the other hand, although sophisticated methods have been developed for fitting complex population genetic models to phylogenies (Volz 2012; Rasmussen, Volz, and Koelle 2014), inference of structural network parameters has to date been limited. However, it has been shown that network structure has a tangible impact on phylogeny shape (Goodreau 2006; Leventhal et al. 2012; Colijn and Gardy 2014; Robinson et al. 2013; Villandre et al. 2016), suggesting that such statistical inference might be possible (Welch, Bansal, and Hunter 2011). In the context of networks, sequence data have the advantage of being objective, in that they are not affected by misreporting. However, just as with survey data, it is important to collect a representative sample from the population to perform accurate inference (Novitsky et al. 2014).

Survey-based studies of sexual networks (Colgate et al. 1989; Liljeros et al. 2001; Schneeberger et al. 2004; Latora et al. 2006; Rothenberg, and Muth, 2007; Clémençon et al. 2015) have found that these networks tend to have a degree distribution which follows a power law [although there has been some disagreement, see (Handcock, and Holland Jones 2004)]. That is, the number of nodes of degree *k* is proportional to $k^{-\gamma }$ for some constant *γ*. When *γ* is in the range 2≤γ≤3, these networks are also referred to as ‘scale-free’ (Barabási, and Albert 1999). One process by which scale-free networks can be generated is preferential attachment (PA), where nodes with a high number of contacts attract new connections at an elevated rate. The first contact network model incorporating PA was introduced by Barabási and Albert (1999), and is now referred to as the Barabási-Albert (BA) model. Under this model, networks are formed by iteratively adding nodes with *m* new edges each. The average degree of the network is therefore 2*m*. In the most commonly studied formulation, these new edges are joined to existing nodes of degree *k* with probability proportional to *k*, so that nodes of high degree tend to attract more connections. BA suggested an extension where the probability of attaching to a node of degree *k* is $k^{\alpha }$ for some non-negative constant *α*, and we use this extension in this work. When α≠1, the degree distribution is no longer a power law: for α<1, the distribution is a stretched exponential, while for α>1, it is a “gelation” type distribution where one or a few hub nodes are connected to nearly every other node in the graph (Krapivsky, Redner, and Leyvraz 2000) (Supplementary Fig. S1).

Previous work offers precedent for the possibility of statistical inference of structural network parameters. Britton and O’Neill (Britton, and O’Neill 2002) develop a Bayesian approach to estimate the edge density in an Erdős-Rényi network (Erdös and Rényi 1960) given observed infection dates, and optionally recovery dates. Their approach was later extended by Groendyke, Welch, and Hunter (Groendyke, Welch, and Hunter 2011) and applied to a much larger data set of 188 individuals. Volz and Meyers (Volz, and Ancel Meyers 2007) and Volz (Volz 2008) developed differential equations describing the spread of a susceptible-infected (SI) epidemic on static and dynamic contact networks with several degree distributions, which could in principle be used for inference if observed incidence trajectories were available. Leigh Brown et al. (2011) analyzed the degree distribution of an approximate transmission network, estimated based on genetic similarity and estimated times of infection, relating 60% of HIV-infected men who have sex with men (MSM) in the United Kingdom. The authors found that a Waring distribution, which is produced by a more sophisticated PA model, was a good fit to their estimated network. However, the transmission network is different from the contact network, containing only those edges which have already led to a new infection. Furthermore, recent experiments have found the correspondence between phylogenetic clusters and network clusters to be weak (Villandre et al. 2016).

Standard methods of model fitting involve calculation of the likelihood of observed data under the model, that is, the probability density of the model having given rise to that data. In maximum likelihood estimation, a quantity proportional to the likelihood is optimized, often through a standard multi-dimensional numerical optimization procedure. Bayesian methods integrate prior information by sampling from the posterior distribution, the product of the prior and the likelihood, instead. To avoid calculation of a normalizing constant, Bayesian inference is often performed using Markov chain Monte Carlo (MCMC), which uses likelihood *ratios* in which the normalizing constants cancel out. Unfortunately, it is generally difficult to explicitly calculate the likelihood of an observed transmission tree under a contact network model, even up to a normalizing constant. To do so, it would be necessary to integrate over all possible networks, and flso over all possible labellings of the internal nodes of the transmission tree (see Supplementary Text S1). A simpler alternative is offered by likelihood-free methods, namely approximate Bayesian computation (ABC) (Tavaré et al. 1997; Beaumont, Zhang, and Balding 2002). ABC leverages the fact that, although calculating the likelihood may be impractical, generating simulated datasets according to a model is often straightforward. If our model fits the data well, the simulated data it produces should be similar to the observed data. More formally, if *D* is the observed data, the posterior distribution f(θ|D) on model parameters *θ* is replaced as the target of statistical inference by $f(\theta |\rho (\hat{D},D)<\epsilon )$, where *ρ* is a distance function, $\hat{D}$ is a simulated dataset according to *θ*, and *ϵ* is a small tolerance (Sunnåker et al. 2013). Typically, *ρ* is chosen to be the difference between one or more summary statistics calculated for each data point. Our group (Poon 2015) and others (Mijung et al. 2015) have demonstrated that a more accurate ABC approximation can be produced by using a kernel function. These functions, popular for machine learning applications, are able to calculate the similarity between data points when considering a very large or even infinite number of data features.

Here, we develop a method using ABC to estimate the parameters of contact network models from observed phylogenetic data. The distance function we use is the tree kernel developed by Poon et al. (2013), which considers all possible subset trees with branch lengths when calculating the similarity between two trees. We apply the method to investigate the parameters of the BA network model on a variety of simulated and real datasets. Our results show that some network parameters can be inferred with reasonable accuracy, while ABC cannot distinguish between different values of others. We also find that these parameters can vary considerably between real epidemics from different settings.

## Methods

### *Netabc*: Phylogenetic Inference of Contact Network Parameters with ABC

We have developed an ABC-based method, *netabc*, to perform statistical inference of contact network parameters from a transmission tree estimated from an observed viral phylogeny. We implemented the adaptive sequential Monte Carlo (SMC) algorithm for ABC developed by Del Moral, Doucet, and Jasra (Del Moral, Doucet, and Jasra 2012). The SMC algorithm keeps track of a population of parameter ‘particles’, which are initially sampled from the parameters’ joint prior distribution. Several datasets are simulated under the model of interest for each of the particles. In this case, the datasets are transmission trees, which are generated by a two-step process. First, a contact network is simulated according to the network model being fit. Second, a transmission tree is simulated over that network with a Gillespie simulation algorithm (Gillespie 1976), in the same fashion as several previous studies (Leventhal et al. 2012; Robinson et al. 2013). Tips of the simulated transmission tree are randomly removed until the simulated tree has the same number of tips as the input tree. The particles are weighted according to the similarity between their associated simulated trees and the observed tree. To quantify this similarity, we used the tree kernel developed by Poon et al. (2013). Particles are iteratively perturbed by applying a Metropolis-Hastings kernel and, if the move is accepted, simulating new datasets under the new parameters. When a particle’s weight drops to zero, because its simulated trees are too dissimilar to the observed tree, the particle is dropped from the population, and eventually replaced by a resampled particle with a higher weight. As the algorithm progresses, the population converges to a Monte Carlo approximation of the ABC target distribution, which is assumed to approximate the desired posterior (Del Moral, Doucet, and Jasra 2012; Sunnåker et al. 2013).

In the original formulation of ABC-SMC (Sisson, Fan, and Tanaka 2007; Beaumont et al. 2009), the user is required to specify a decreasing sequence of tolerances $\left\{\epsilon_{i}\right\}$. At iteration *i*, particles with no associated simulated datasets within distance $\epsilon_{i}$ of the observed data are removed from the population. In the adaptive version of Del Moral, Doucet, and Jasra (2012), the sequence of tolerances is determined automatically by fixing the decay rate of the population’s effective sample size (ESS) to a user-defined value. Del Moral, Doucet, and Jasra (Del Moral, Doucet, and Jasra 2012) call this value *α*, but we will refer to it here as $\alpha_{\text{ESS}}$ to avoid confusion with the PA power parameter of the BA model.

To check that our implementation of Gillespie simulation was correct, we reproduced Figure 1A of Leventhal et al. (2012) (our Supplementary Fig. S2), which plots the imbalance of transmission trees simulated over four network models at various levels of pathogen transmissibility. Our implementation of adaptive ABC-SMC was tested by applying it to the same mixture of Gaussians used by Del Moral, Doucet, and Jasra (2012) to demonstrate their method (originally used by Sisson, Fan, and Tanaka 2007). We were able to obtain a close approximation to the function (see Supplementary Fig. S3), and attained the stopping condition used by the authors in a comparable number of steps.

**Figure 1.**
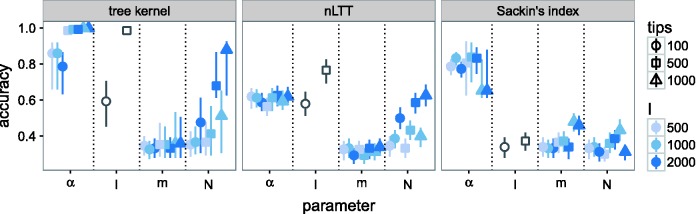
Cross-validation accuracy of kernel-SVM classifier (left), SVM classifiers using nLTT (centre) and Sackin's) index (right) for BA model parameters. Kernel meta-parameters were set to λ=0.3 and *σ* = 4. Each point was calculated based on 300 simulated transmission trees over networks with three different values of the parameter being tested, assuming perfect knowledge of the other parameters. Vertical lines are empirical 95% CIs based on 1,000 2-fold cross-validations. The classifiers for *I* were not evaluated with 1,000-tip trees, because one of the tested *I* values was 500, and it is not possible to sample a tree of size 1,000 from 500 infected individuals.

Nodes in our networks followed simple SI dynamics, meaning that they became infected at a rate proportional to their number of infected neighbours, and never recovered. For all analyses, the transmission trees’ branch lengths were scaled by dividing by their mean. We used the *igraph* library’s implementation of the BA model (Csardi, and Nepusz 2006) to generate the graphs. The analyses were run on Westgrid (https://www.westgrid.ca/) and a local computer cluster.

### Classifiers for BA Model Parameters from Tree Shapes

We considered four parameters related to the BA model, denoted *N*, *m*, *alpha*, and *I*. The first three of these parameterize the network structure, while *I* affects the simulation of transmission trees over the network. However, we will refer to all four as BA parameters. *N* denotes the total number of nodes in the network, or equivalently, susceptible individuals in the population. *m* is the number of new undirected edges added for each new vertex, or equivalently one-half of the average degree. *α* is the power of PA—new nodes are attached to existing nodes of degree *d* with probability proportional to $d^{\alpha }+1$. Finally, *I* is the number of infected individuals at the time when sampling occurs. The *α* parameter is unitless, while *m* has units of edges or connections per vertex, and *N* and *I* both have units of nodes or individuals.

Before proceeding with a full validation of *netabc* on simulated data, we undertook an experiment designed to assess whether different values of these parameters could be distinguished from tree shapes; in other words, the parameters’ identifiability. One parameter of the BA model was investigated at a time while holding all others fixed, a strategy commonly used when performing sensitivity analyses of mathematical models. This allowed us to perform a fast preliminary analysis without dealing with the ‘curse of dimensionality’ of the full parameter space. We simulated trees under three different values of each parameter, and asked how well we could tell the different trees apart. The better we are able to distinguish the trees, the more identifiability we might expect for the corresponding parameter when we attempt to estimate it with ABC.

This experiment also had the secondary purpose of validating our choice of the tree kernel as a distance measure in ABC. To tell the trees apart, we used a classifier based on the tree kernel, but we also tested two other tree shape statistics. Sackin’s index (Shao 1990) is a measure of tree imbalance which not take branch lengths into account, considering only the topology. The normalized lineages-through-time [nLTT, Janzen, Höhna, and Etienne (2015)] compares two trees based on normalized distributions of their branching times, and does not explicitly consider the topology. Since the tree kernel incorporates both of these sources of information, we expected it to outperform the other two statistics. Finally, the tree kernel can be tuned by adjusting the values of the meta-parameters *λ* and *σ* (the ‘decay factor’ and ‘radial basis function variance’, see Poon et al. Poon et al. 2013). *λ* is used to penalize large subset trees which tend to dominate the kernel score. When *λ*  =  0, all but the root branches of each subset tree are ignored, while when λ=1, no penalty is applied. *σ* controls how strictly the notion of similarity is applied to branch lengths. When *σ* = 0, branch lengths must match exactly, while as σ→∞, branch lengths are not considered at all.

We simulated 100 networks under each of three different values of *α*: 0.5, 1.0, and 1.5 (300 networks total). The other parameters were fixed to the following values: *N* = 5000, *I* = 1000, and *m* = 2. A transmission tree with 500 tips was simulated over each network (300 transmission trees total). The 300 trees were compared pairwise with the tree kernel to form a 300 × 300 kernel matrix. The kernel meta-parameters *λ* and *σ* were set to 0.3 and 4 respectively. We also computed a 300 × 300 matrix of pairwise nLTT values, and a 1 × 300 vector of Sackin's index values. We constructed three classifiers for *α*: a kernel support vector regression (kSVM) from the kernel matrix with the *kernlab* package (Zeileis et al. 2004) and two ordinary SVMs from the nLTT matrix and Sackinry index vector with the *e1071* package (Meyer 2015). The accuracy of each classifier was evaluated with 1,000 2-fold cross validations with equally sized folds.

Three similar experiments were performed for the other BA model parameters (one experiment per parameter). *m* was varied between 2, 3, and 4; *I* between 500, 1,000, and 2,000; and *N* between 3,000, 5,000, and 8,000. The parameters not being tested were fixed at the values *N* = 5000, *I* = 1000, *m* = 2, and *α* = 1. Thus, we performed a total of four cross-validations for each classifier, one for each of the BA model parameters *α*, *I*, *m*, and *N*. We repeated these four cross-validations with different values of *λ* (0.2, 0.3, and 0.4) and *σ* ($2^{-3},\;2^{-2}$, … , 2^3^), as well as on trees with differing numbers of tips (100, 500, and 1,000). For the structural parameters *α*, *m*, and *N*, the experiments were repeated with three different fixed values of *I* (500, 1,000, and 2,000). The combination of the number of sampled individuals (*i.e.* the number of tips) and the epidemic size (i.e. *I*) will be referred to as an “epidemic scenario”. When evaluating the classifier for *I*, we did not consider trees with 1,000 tips, because one of the tested *I* values was 500, and the number of tips cannot be larger than *I*.

### ABC Simulations

We tested *netabc* by jointly estimating the four parameters of the BA model. We used the standard validation approach of simulating transmission trees under the model with known parameter values and attempting to recover those values with *netabc*. The algorithm was not informed of any of the true parameter values for the main set of simulations. We simulated three transmission trees, each with 500 tips, under every element of the Cartesian product of these parameter values: *N* = 5,000, *I* = {1,000, 2,000}, *m* = {2, 3, 4}, and *α* = {0.0, 0.5, 1, 1.5}. This produced a total of 24 parameter combinationsombihree trees per combination = 72 trees total. The adaptive ABC algorithm was applied to each tree with these priors: m∼ DiscreteUniform(1, 5), α∼ Uniform(0, 2), and (*N*, *I*) jointly uniform on the region {500≤N≤15,000, 500≤I≤5,000, I≤N}. Proposals for *α*, *N*, and *I* were Gaussian, while proposals for *m* were Poisson. Following Del Moral, Doucet, and Jasra (Del Moral, Doucet, and Jasra 2012) and Beaumont et al. (2009), the variance of all proposals was equal to the empirical variance of the particles.

The SMC settings used were 1000 particles, 5 simulated datasets per particle, and $\alpha_{\text{ESS}}=0.95$. We used the same stopping criterion as Del Moral, Doucet, and Jasra (2012), namely when the MCMC acceptance rate dropped below 1.5%. Approximate posterior means for the parameters were obtained by taking the weighted average of the final set of particles. Highest posterior density (HPD) intervals were calculated with the *HPDinterval* function from the *R* package *coda* (Plummer et al. 2006).

To evaluate the effects of the true parameter values on the accuracy of the posterior mean estimates, we analyzed the *α* and *I* parameters individually using generalized linear models (GLMs) The response variable was the error of the point estimate, and the predictor variables were the true values of *α*, *I*, and *m*. We did not test for differences across true values of *N*, because *N* was not varied in these simulations. The distribution family and link function for the GLMs were Gaussian and inverse, respectively, chosen by examination of residual plots and Akaike information criteria (AIC). The *P*-values of the estimated GLM coefficients were corrected using Holm-Bonferroni correction (Sture, 1979) with *n* = 6 (two GLMs with three predictors each). Because there was clearly little to no identifiability of *N* and *m* with ABC (see results in next section), we did not construct GLMs for those parameters.

Two further simulations were performed to address the possible impact of two types of model misspecification. To evaluate the effect of model misspecification in the case of heterogeneity among nodes, we generated a network where half the nodes were attached with power *α* = 0.5, and the other half with power *α* = 1.5. The other parameters for this network were *N* = 5,000, *I* = 1000, and *m* = 2. To investigate the effects of potential sampling bias, we simulated a transmission tree where the tips were sampled in a peer-driven fashion, rather than at random. That is, the probability to sample a node was twice as high if any of that nodeee network peers had already been sampled. The parameters of this network were *N* = 5,000, *I* = 2,000, *m* = 2, and α=0.5.

Despite the fact that the parameter values used to generate the simulated transmission trees were known, the true posterior distributions of the BA parameters were unknown. Therefore, any apparent errors or biases in the estimates could be due to either poor performance of our method, or to real features of the posterior distribution. Two retrospective experiments were performed to disambiguate some of the observed errors. To assess the impact of the SMC settings on *netabc*’s accuracy, we ran *netabc* twice on the same simulated transmission tree. For the first run, the SMC settings were the same as in the other simulations: 1,000 particles, 5 simulated transmission trees per particle, and $\alpha_{\text{ESS}}=0.95$. The second run was performed with 2,000 particles, 10 simulated transmission trees per particle, and $\alpha_{\text{ESS}}=0.97$. To investigate the extent to which errors in the estimated BA parameters were due to true features of the posterior, rather than an inaccurate ABC approximation, we performed marginal estimation for one set of parameter values. Each combination of 1, 2, or 3 model parameters (14 combinations total) was fixed to their known values, and the remaining parameters were estimated with *netabc*. The parameter values were α=0.0, *m* = 2, *I* = 2,000, and *N* = 5,000.

### Investigation of Published Data

We applied our ABC method to ten published HIV datasets. Because the BA model generates networks with a single connected component, we specifically searched for datasets which originated from existing clusters, either phylogenetically or geographically defined. Characteristics of the datasets we investigated are given in Table 1. For clarity, we will refer to each dataset by its risk group and location of origin in the text. For example, the Zetterberg et al. (2004) data will be referred to as IDU/Estonia.

**Table 1.** Characteristics of published datasets investigated with ABC

Reference	Sequences (*n*)	Location	Risk group	Gene
Zetterberg et al. (2004)	171	Estonia	IDU	*env*
Niculescu et al. (2015)	136	Romania	IDU	*pol*
Novitsky et al. (2013)	180	Mochudi, Botswana	HET	*env*
Novitsky et al. (2014)				
McCormack et al. (2002)	141/154	Karonga District, Malawi	HET	*env*/*gag*
Grabowski et al. (2014)	225	Rakai District, Uganda	HET	*env*/*gag*
Wang et al. (2015)	173	Beijing, China	MSM	*pol*
Kao et al. (2011)	275	Taiwan	MSM	*pol*
Little et al. (2014)	180	San Fransisco, USA	MSM	*pol*
Li et al. (2015)	280	Shanghai, China	MSM	*pol*
Cuevas et al. (2009)	287	Basque Country, Spain	mixed	*pol*

Acronyms: MSM, men who have MSM; IDU, injection drug users; HET, heterosexual. The HET data were sampled from a primarily heterosexual risk environment, but did not explicitly exclude other risk factors. The sequences column indicates how many sequences were included in our analysis; there may have been additional sequences linked to the study which we excluded for various reasons (see ‘Methods’ section).

We downloaded all sequences associated with each published study from GenBank. For the IDU/Romania data, only sequences from injection drug users (IDU, whose sequence identifiers included the letters “DU”) were included in the analysis. Kao et al. (2011) (MSM/Taiwan) found a strong association in their study population between subtype and risk group - subtype B was most often associated with men who have MSM, whereas IDU were usually infected with a circulating recombinant form. Since there were many more subtype B sequences in their data than sequences of other subtypes, we restricted our analysis to the subtype B sequences and labelled this dataset as MSM. Two datasets (HET/Uganda and HET/Malawi) included both *env* and *gag* sequences. Each gene was analyzed separately to assess the robustness of *netabc* to the particular HIV gene sequence used to estimate a transmission tree. The IDU/Estonia data also sequenced both genes, but the highly variable coverage and high homology of the *gag* sequences made it impossible to obtain a sufficiently large block of non-identical sequences to analyze. Therefore, we analyzed only *env* for this dataset.

Each *env* sequence was aligned pairwise to the HXB2 reference sequence (GenBank accession number K03455), and the hypervariable regions were clipped out with *BioPython* version 1.66+ (Cock et al. 2009). Sequences were multiply aligned using *MUSCLE* version 3.8.31 (Edgar 2004), and alignments were manually inspected with *Seaview* version 4.4.2 (Gouy, Guindon, and Gascuel 2010). Duplicated sequences were removed with *BioPython*; the number of these duplicates was between 0 and 7 for all but the IDU/Estonia data, from which 21 sequences were removed. Phylogenies were constructed from the nucleotide alignments by approximate maximum likelihood using *FastTree2* version 2.1.7 (Price, Dehal, and Arkin 2010) with the generalized time-reversible (GTR) model (Tavaré 1986). Transmission trees were estimated by rooting and time-scaling the phylogenies by root-to-tip regression, using a modified version of *Path-O-Gen* (distributed as part of *BEAST* (Drummond and Rambaut 2007)) as described previously in Poon (2015). All estimated transmission trees were fully binary.

To check if our results were robust to the choice of phylogenetic reconstruction method, we built and reanalyzed phylogenies for the datasets with the lowest and highest estimated *α* values (mixed/Spain and IDU/Estonia) with *RAxML* (Stamatakis 2014) with the GTR + Γ model of sequence evolution and rate heterogeneity. The trees were rooted and time-scaled with *Least Squares Dating* (LSD) (To et al. 2016). For expediency, the analysis was run with the prior m∼DiscreteUniform(2,5), which defines a smaller total search space than the prior allowing *m* = 1. For both of these datasets, we also analyzed five bootstrap replicate alignments generated by resampling alignment columns with replacement. For the dataset with the largest number of duplicated sequences (IDU/Estonia; 21 sequences), we repeated the analysis without removing the duplicates.

Four of the datasets (MSM/Shanghai, HET/Botswana, HET/Uganda, and MSM/USA) were initially much larger than the others, containing 1,265, 1,299, 1,026/915 (*env*/*gag*), and 648 sequences respectively. To ensure that the analyses were comparable, we reduced these to a number of sequences similar to the smaller datasets. For the MSM/Shanghai data, we detected a cluster of size 280 using a patristic distance cutoff of 0.02 as described previously in Stamatakis (2014). Only sequences within this cluster were carried forward. For the HET/Uganda, HET/Botswana, and MSM/USA data, no large clusters were detected using the same cutoff, so we analyzed subsets of sizes 255, 180, and 180, respectively. The subset of the HET/Uganda data was chosen by eye such that the individuals were monophyletic in both the *gag* and *env* trees. The other subsets were arbitrarily chosen subtrees from phylogenies of the complete datasets.

For all datasets, we used the priors *α* ∼ Uniform(0, 2) and *N* and *I* jointly uniform on the region {n≤N≤10,000, n≤I≤10,000, I≤N}, where *n* is the number of tips in the tree. Since the value *m* = 1 produces networks with no cycles, which we considered fairly implausible, we ran one analysis with the prior m∼ DiscreteUniform(1, 5), and one with the prior m∼ DiscreteUniform(2, 5). The other parameters to the SMC algorithm were the same as used for the simulation experiments, except that we used 10,000 particles instead of 1,000 to increase the accuracy of the estimated posterior for all analyses except the bootstrap replicates. This was computationally feasible due to the small number of runs required for this analysis.

## Results

### Classifiers for BA Model Parameters from Tree Shapes

We investigated the identifiability of four parameters of the BA network model (Barabási and Albert 1999): the number of nodes *N*, the PA power *α*, the number of edges added per vertex *m*, and the number of infected nodes *I*. To examine the effect of these parameters on tree shape, we simulated transmission trees under different parameter values, calculated pairwise tree kernel scores between them, and attempted to classify the trees using a kSVM. We also tested classifiers based on Sackin's index (Shao 1990) and the nLTT statistic (Janzen, Höhna, and Etienne 2015). We report the accuracy of the classifiers, which is simply the proportion of trees which were assigned the correct parameter value. Since there were three possible values, random guessing would produce an accuracy of 0.33. The results are shown in fig. 1. Classifiers based on the nLTT and Sackin's index generally exhibited worse performance than the tree kernel, although the magnitude of the disparity varied between the parameters (Fig. 1, centre and right). Larger datasets were generally classified more accurately (Supplementary Figs. S4–S7), although large values of *λ* produced worse estimates on large datasets. Extremely low *σ* values, which require nearly-exact matches between branch lengths, resulted in low accuracy in some cases (e.g. Supplementary Fig. S4, center row).

The kSVM classifier for *α* had an average accuracy of 0.92, compared with 0.6 for the nLTT, and 0.77 for Sackin's index. No classifier could accurately identify *m* in any epidemic scenario, with average accuracy values of 0.35 for kSVM, 0.32 for the nLTT, and 0.38 for Sackin's index. There was little variation in accuracy between epidemic scenarios, although the accuracy of the kSVM was slightly higher on 1,000-tip trees (Fig. 1, left).

The accuracy of classifiers for *I* varied significantly with the number of tips in the tree. For 100-tip trees, the average accuracy was 0.59, 0.58, and 0.34 for the tree kernel, nLTT, and Sackin's index, respectively. For 500-tip trees, the values increased to 0.99, 0.76, and 0.37. Finally, the performance of classifiers for *N* depended heavily on the epidemic scenario. The accuracy of the kSVM classifier ranged from 0.36 for the smallest epidemic and smallest sample size, to 0.81 for the largest. Accuracy for the nLTT ranged from 0.33 to 0.63. Sackin's index did not accurately classify *N* in any scenario, with an average accuracy of 0.35 and little variation between scenarios.

### ABC Simulations

**Figure 2.**
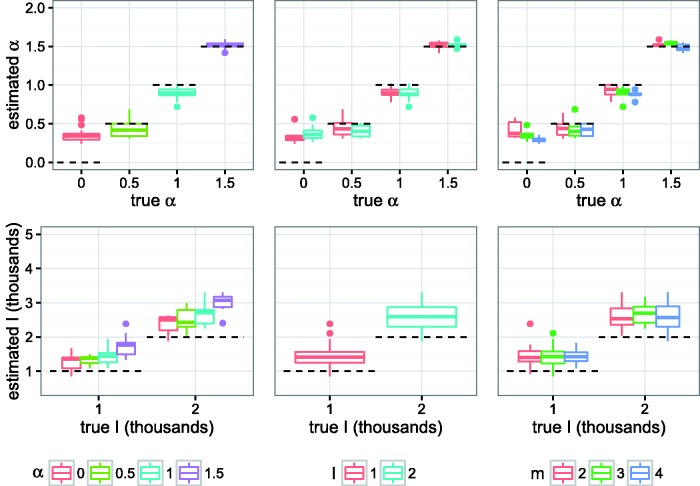
Posterior mean point estimates for BA model parameters *α* and *I* obtained by running *netabc* on simulated data, stratified by true parameter values. First row of plots contains true vs. estimated values of *α*; second row contains true vs. estimated values of *I*. Columns are stratified by *α*, *I*, and *m*, respectively. Dashed lines indicate true values.

Figure 2 shows stratified posterior mean point estimates of the BA model parameters *α* and *I*, obtained with ABC on simulated data. The parameters *m* and *N* were not identifiable with ABC for any parameter combinations (Supplementary Fig. S8). Average boundaries of 95% HPD intervals for all parameters are given in Table 2.

**Table 2.** Average posterior mean point estimates and 95% HPD interval widths for BA model parameter estimates obtained with *netabc* on simulated data

Parameter	True value	Mean point estimate	Mean HPD lower bound	Mean HPD upper bound
*α*	0.0	0.36	0.01	0.81
	0.5	0.43	0.04	0.83
	1.0	0.90	0.51	1.09
	1.5	1.52	1.26	1.81
*I*	1000	1450	651	2,592
	2000	2622	1114	4,080
*m*	2	2.96	2.00	5.00
	3	3.04	2.04	4.96
	4	3.17	1.88	5.00
*N*	5,000	9,041	2,613	14,659

Three transmission trees were simulated under each combination of the listed parameter values, and the parameters were estimated with ABC without training.

Across all simulations, the median [IQR] absolute errors of the parameter estimates obtained with *netabc* were 0.11 [0.03–0.25] for *α*, 492 [294–782] for *I*, 1 [0–1] for *m*, and 4,153 [3,660–4,489] for *N*. These errors comprised, respectively, 6%, 11%, 17%, and 29% of the regions of nonzero prior density. For *I* and *N*, relative errors were 38% [20–50%] and 83% [73–90%]. Average 95% HPD interval widths were 0.68, 2,454, 3.01, and 12,046, representing 34, 55, 50, and 83% of the nonzero prior density regions. Point estimates of *I* were upwardly biased: *I* was overestimated in 69 out of 72 simulations (96%). The estimates for *m* and *N* were similar across all simulations (median [IQR] point estimates 3 [3–3] and 9,153 [8,660–9,489]) regardless of the true values of any of the BA parameters (Supplementary Fig. S8).

To analyze the effects of the true parameter values on the accuracy our estimates of *α* and *I*, we fitted one GLM for each of these two parameters, with error rate as the dependent variable and the true parameter values as independent variables. Since the estimates of *m* and *N* were roughly equal across all simulations (Supplementary Fig. S8), GLMs were not fitted for these parameters. The estimated coefficients are shown in Table 3. The GLM analysis indicated that the error in estimates of *α* decreased with larger true values of *α* ($P<10^{-5}$) and *m* (*P* = 0.01) but was not significantly affected by *I*. Qualitatively, *α* seemed to be only weakly identifiable between the values of 0 and 0.5 (Fig. 2). The error in the estimated *I* value was slightly lower for smaller values of *α* ($P<10^{-5}$) and *I* (*P* = 0.05), but was not significantly affected by the true value of *m*.

**Table 3.** Parameters of fitted GLMs relating error in estimated *α* and *I* to true values of BA parameters

Dependent variable	Independent variable	Estimate	Standard error	*P*-value
*α* error	(Intercept)	2	0.6	0.01
	true *α*	10	2	$<10^{-5}$
	true *I*	$-3\times 10^{-4}$	$2\times 10^{-4}$	0.7
	true *m*	0.5	0.2	0.01
*I* error	(Intercept)	0.004	$5\times 10^{-4}$	$<10^{-5}$
	true *α*	–0.001	$2\times 10^{-4}$	$<10^{-5}$
	true *I*	$-4\times 10^{-7}$	$2\times 10^{-7}$	0.05
	true *m*	$-7\times 10^{-5}$	$8\times 10^{-5}$	1

GLMs ere fitted with the Gaussian distribution and inverse link function. Coefficients are interpretable as additive effects on the inverse of the mean error.

**Figure 3.**
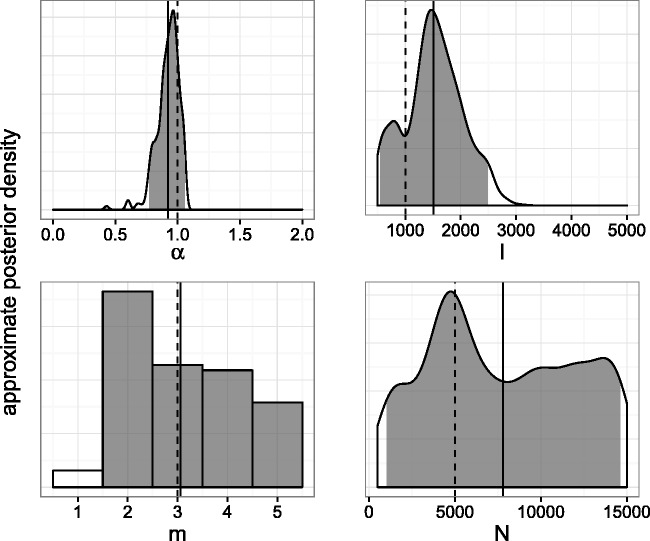
One-dimensional marginal posterior distributions of BA model parameters estimated by *netabc* from a simulated transmission tree. Dashed lines indicate true values, solid lines indicate posterior means, and shaded areas show 95% HPD intervals.

**Figure 4.**
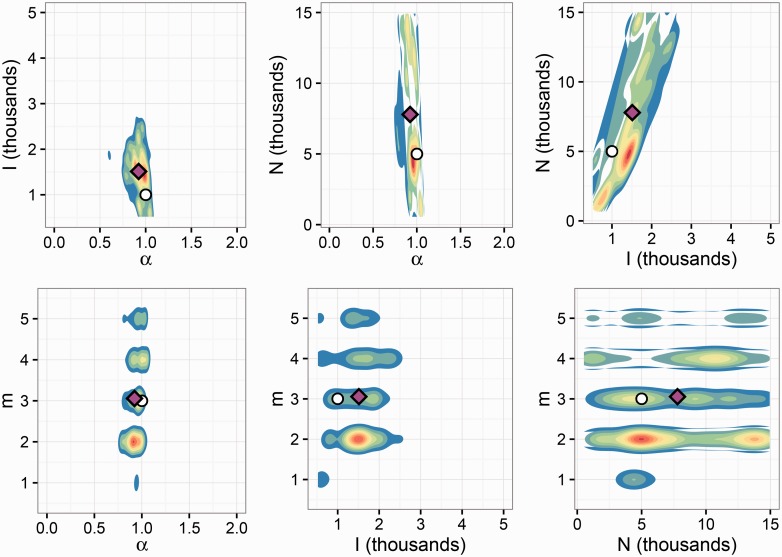
Two-dimensional marginal posterior distributions of BA model parameters estimated by *netabc* from a simulated transmission tree. White circles indicate true values, magenta diamonds indicate posterior means. Marginals in *m* (bottom row) have non-zero density only at integral values of *m*; the appearance of density at non-integral values for is visualization only.

The dispersion of the ABC approximation to the posterior also varied between the parameters (Table 2). HPD intervals around *α* and *I* were often narrow relative to the region of nonzero prior density, whereas the intervals for *m* and *N* were more widely dispersed. Figures 3 and 4 show one- and two-dimensional marginal distributions for a simulation with *α* and *I* errors close to their respective medians. The parameters for this simulation were *α* = 1, *I* = 1,000, *m* = 3, and *N* = 5,000. The 2D marginals indicate some dependence between pairs of parameters, particularly *I* and *N* which show a diagonally shaped region of high posterior density.

To test the effect of model misspecification, we simulated one network where the nodes exhibited heterogeneous PA power (half 0.5, the other half 1.5), with *m* = 2, *N* = 5,000, and *I* = 1,000. The posterior mean [95% HPD] estimates for each parameter were: *α*, 1.03 [0.67–1.18]; *I*, 1,474 [511–2,990]; *m*, 3 [1–5]; *N*, 9,861 [3,710–14,977]. To test the effect of sampling bias, we sampled one transmission tree in a peer-driven fashion, where the probability to sample a node was twice as high if one of its peers had already been sampled. The parameters for this experiment were *N* = 5,000, *m* = 2, *α* = 0.5, and *I* = 2,000. The estimated values were *α*, 0.3 [0–0.63]; *I*, 2,449 [1,417–3,811]; *m*, 3 [2–5]; *N*, 9,132 [2,852–14,780]. Both of these results were in line with estimates obtained on other simulated datasets (Table 2), although the estimate of *α* for peer-driven sampling was somewhat lower than typical.

Figure S9 shows the effect of performing marginal ABC estimation of each of the BA parameters on the same simulated transmission tree. The estimates of *m* were apparently unaffected by marginalizing out the other parameters, corroborating the previous experiments’ findings that *m* is not an identifiable parameter from scaled tree shapes. Compared to allowing all parameters to vary, estimates of *α*, *I*, and *N* were improved by 41, 59, and 46% when all other parameters were fixed. Figure S10 shows the impact of increasing the number of particles, simulated datasets, and $\alpha_{\text{ESS}}$ parameter on the accuracy of a single simulation. The number of iterations until the stopping condition was reached was 81 with the basic settings and 124 with the higher settings. The results of the two simulations were similar, but surprisingly, the results with higher SMC settings were slightly worse (by 10, 8, and 11% for *α*, *I*, and *N*, respectively). However, the 50% HPD interval for *I* was closer to the true value of 2,000 with the improved settings (2,338–3,423, vs. 2,810–3,767 with basic settings). The estimate of *m*, 3 in both cases, was unaffected by the settings.

### Published HIV Data

**Figure 5.**
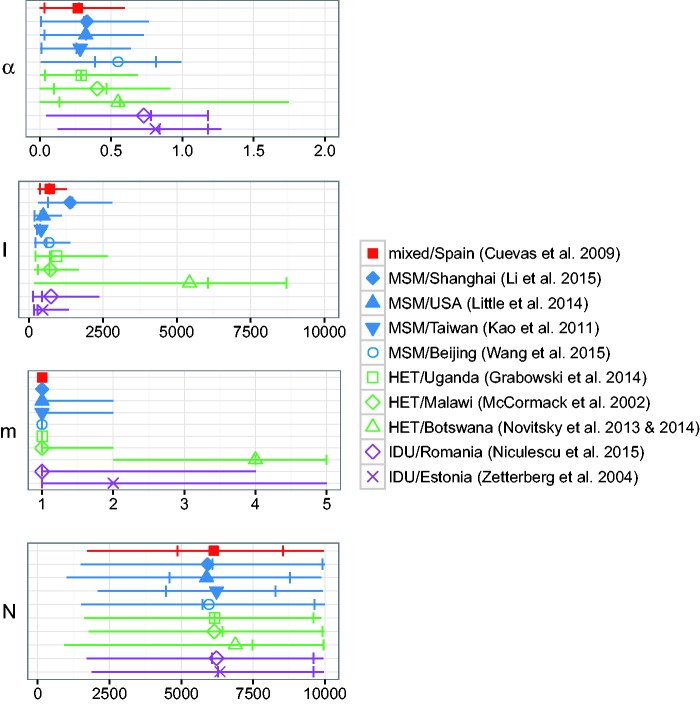
Posterior means (points), 50% HPD intervals (notches), and 95% HPD intervals (lines) for parameters of the BA network model, fitted to ten HIV datasets with *netabc*. Legend labels indicate risk group and country of origin. Abbreviations: IDU, injection drug users; MSM, men who have MSM; HET, heterosexual. Note that posterior means can fall outside of the HPD interval if the distribution is diffuse.

We applied ABC to five published HIV datasets (Table 1), and found substantial heterogeneity among the parameter estimates (Fig. 5 and Supplementary Fig. S11). Posterior mean point estimates and 50% and 95% HPD intervals for each parameter are shown in Figure 5. Supplementary Figure S11 shows point estimates and HPD intervals obtained when the value *m* = 1 was disallowed by the prior. Since the results indicated that *m* = 1 was the most credible value for several datasets, all results discussed henceforth apply to the prior m∼DiscreteUniform(1,5) unless otherwise stated.

Posterior mean point estimates for the PA power *α* were all sub-linear, ranging from 0.27 (mixed/Spain) to 0.83 (IDU/Estonia). When aggregated by risk group, the average estimates were 0.78 for IDU, 0.41 for primarily heterosexual risk, and 0.37 for MSM. 95% HPD intervals were very wide for most datasets, often encompassing nearly the interval from 0 to 1 (Fig. 5). As shown in Supplementary Figure S12, the estimates of *α* were quite robust to the gene analyzed.

For all but the HET/Botswana data, the posterior mean estimates for *I* were between 373 (IDU/Estonia) and 1391 (MSM/Shanghai). The HET/Botswana data had a much higher estimated *I* value (5,432) than the other datasets, with a very wide 95% HPD interval covering almost the entire prior region (Fig. 5). There was no significant correlation between the number of sequences in the tree and the estimated *I* value (Spearman correlation, *P* = 0.9), indicating that the higher estimates were not simply due to increased sampling density. When both *gag* and *env* sequences were analyzed, the estimates from the *env* data were higher (HET/Uganda, 939 for *gag* vs. 1615 for *env*; HET/Malawi, 724 for *gag* vs. 845 for *env*).

The posterior means of *m* were equal to one for eight of the datasets analyzed. The widths of the 95% HPD intervals varied from 0 (all the mass on the estimated value) to 5 (the entire prior region). Estimates of *N* were mostly uninformative, with very similar estimates for all datasets (mean 6,202, range 5,881–6,882). This was similar to the pattern observed for the synthetic data, where the posterior mean always fell around the upper two-thirds mark of the range (fig. S8).

When the value *m* = 1 was disallowed by the prior, the separation in *α* between the IDU datasets and the others became more striking (Supplementary Fig. S11). Both IDU datasets had estimated *α* values at or above 1. The estimate for the MSM/Beijing data was slightly lower (0.85) and the estimates for the seven remaining non-IDU datasets were bounded above by 0.58. The values of *I* were fairly robust to the choice of prior (compare Fig. 5 and Supplementary Fig. S11), although the 95% HPD intervals were slightly narrower (average width 2,159 for m≥1 and 1,874 for m≥2). The posterior means of *m* for all but the HET/Botswana data took on the value 3 with this prior, with the HPD intervals spanning the entire prior region. This is very similar to the results observed for *m* on simulated data (Table 2), and suggests that *m* is not identifiable from these data with this prior. The results for *N* did not change appreciably between the two choices of prior.

For the two datasets we reanalyzed using RAxML (Stamatakis 2014) and LSD (To et al. 2016), *α* was relatively robust to the choice of method (fig. S13, posterior means 0.48 vs. 0.48 for mixed/Spain and 1.02 vs. 1.12 for IDU/Estonia). However, the estimates of *I* were about twice as high when RAxML was used instead of FastTree to reconstruct the trees (228 vs. 437 for IDU/Estonia, 816 vs. 1,949 for mixed/Spain). Supplementary Figure S14 shows estimates obtained for five bootstrap replicate alignments for each of these two datasets. For the mixed/Spain data, the estimated posterior mean [range of bootstrap posterior means] was 0.48 [0.54–0.67] for *α*, 816 [403 - 886] for *I*, 2.76 [2.61–3.24] for *m*, and 6,639 [6,652–7245] for *N*. For the IDU/Estonia data, values were 1.02 [0.78–1.07] for *α*, 228 [313–741] for *I*, 3.11 [3.41–3.46] for *m*, and 6,803 [5,941–6,913] for *N*. When duplicated sequences were included in the analysis of the IDU/Estonia data, the posterior means were α=0.88 (vs. 0.83 with duplicates excluded), *I* = 707 (vs. 373), *m* = 1.53 (vs. 1.72), and *N* = 6,517 (vs. 6,486).

## Discussion

Contact networks can have a strong influence on epidemic progression, and are potentially useful as a public health tool (Little et al. 2014; Wang et al. 2015). Despite this, few methods exist for investigating contact network parameters in a phylodynamic framework [although see Leventhal et al. 2012, Groendyke et al. 2011, Volz 2009, and Leigh Brown et al. 2011 for related work]. ABC is a model-agnostic method which can be used to investigate any quantity that affects tree shape (Poon 2015). In this work, we developed a ABC-based method to infer the parameters of a contact network model. The method is general, and could be applied to any model from which contact networks can be simulated. We demonstrated the method on the BA model, which is a simple model incorporating a ‘rich get richer’, or PA, partner formation mechanism. For some parameter choices, the BA model gives rise to the power law degree distributions commonly observed in real-world networks.

### Analysis of BA Model with Synthetic Data

The *α* parameter of the BA model, known as the PA power, controls how strongly new nodes are attracted to existing nodes of higher degree. This parameter had a strong influence on tree shape in the range of values we considered. Although the tree kernel was the most effective classifier for *α*, Sackin's index, a measure of tree imbalance, performed nearly as well (Fig. 1). High *α* values produce networks with few well-connected “superspreader” nodes which are involved in a large number of transmissions, resulting in a highly unbalanced ladder-like tree structure. There appeared to be weaker identifiability for α<1 than for α≥1 (Fig. 2 and Table 2), meaning that values below 1 were less distinguishable from each other based on tree shape alone. This observation may be partially explained by the relationship between *α* and the exponent *γ* of a power law fitted to the networkts degree distribution (Supplementary Fig. S15). Although the degree distributions do not truly follow a power law for α≠1, the fitted exponent still captures the shape of the degree distribution reasonably well (Supplementary Fig. S1). The *γ* values fitted to *α* = 0 and α=0.5 are nearly identical (about 2.28 for *α* = 0 and 2.33 for α=0.5 with *N* = 5000 and m=2). In other words, the degree distributions of networks with α<1 are similar to each other, which may result in similarity of corresponding transmission trees as well.

*I*, representing the number of infected individuals at the time of sampling, was also identifiable, albeit over-estimated with ABC for both values we considered. Sackin's index was better able to discern *I* from tree shape than the nLTT (Fig. 1 and Supplementary Fig. S6), suggesting that this parameter impacts the distribution of branching times in the tree more than the topology. In a homogeneously-mixed population, branching times can be modelled by the coalescent process (Kingman 1982), in our case under the SI model (Volz et al. 2009). Although networks are not homogeneously mixed, the forces which affect the distribution of branching times still apply. In our simulations, all edges shared the same transmission rate, so that the waiting time until the next transmission in the entire network was always inversely proportional to the number of edges between infected and uninfected individuals. In the initial phase of the epidemic, when *I* is small, each new transmission results in many such edges. Hence, there is an early exponential growth phase, producing many short branches near the root of the tree. As the epidemic gets closer to saturating the network, the number of these edges decays, causing longer waiting times.

The number of nodes in the network, *N*, exhibited the most variation in terms of its effect on tree shape. There was almost no measurable difference between trees simulated under different *N* values when the number of infected nodes *I* was small (Supplementary Fig. S7). In retrospect, it is unreasonable to expect good estimation of *N*, in many cases, because adding additional nodes does not change the edge density or overall shape of a BA network. This can be illustrated by imagining that we add a small number of nodes to a network after the epidemic simulation has already been completed. If *I* is small relative to *N*, very few of the infected nodes will gain any new neighbours. Thus, the outcome of a second simulation on the same network will likely be very similar. On the other hand, when *I* is large relative to *N*, the coalescent dynamics discussed above also apply. The waiting times until the next infection increase, resulting in longer coalescence times toward the tips. The relative accuracy of the nLTT in these situations (Fig. 1 and Supplementary Fig. S7) corroborates this hypothesis, as the nLTT uses only information about the coalescence times. When all BA parameters were simultaneously estimated with ABC, *N* was nearly always over-estimated by approximately a factor of two (Supplementary Fig. S8 and Fig. 2). One factor which may have contributed to this bias was our choice of prior distribution. Since the prior for *I* and *N* was jointly uniform on a region where I≤N, more prior weight was assigned to higher *N* values. We note also that this prior places more mass on low *I* values. However, the estimate of *I* was very high for the HET/Botswana data, suggesting that a strong enough signal in the data can overcome the prior. Furthermore, when *I* was estimated marginally with fixed *N*, the accuracy of the estimate improved even though there was no longer any extra prior mass on low *I* values.

Another possible contributing factor to the overestimation of *I* and *N* relates to the dynamics of the SI model and the coalescent process. The number of infected individuals follows a logistic growth curve under the SI model. This kind of growth curve has three qualitative phases: exponential growth, linear growth, and a slow final phase when the susceptible population is almost depleted. The waiting times until the next transmission, which determine the coalescence times in the tree, are dependent on the growth phase of the epidemic. Therefore, we hypothesize that it is the growth phase at the time of sampling which most affects tree shape, rather than the specific values of *I* or *N* (Supplementary Fig. S17). As shown in Supplementary Figure S16, there are contiguous values of *I* and *N* for which both derivatives are similar If *N* and *I* are free to vary (as is the case in ABC), both parameters may be overestimated. We also note the resemblance of the contour surface of Supplementary Figure S16 to the 2D marginal posterior distribution on *I* and *N* obtained with simulated data (Fig. 4).

The *m* parameter, which controls the number of connections added to the network per vertex, did not have a measurable impact on tree shape and was not identifiable with ABC. It was pointed out to us by an anonymous reviewer that for a fixed *I*, an infected node may only end up transmitting along a fraction of its outgoing edges, which could mask the presence of the extra edges associated with higher *m*. If *m* were parameterized by a continuous variable, it is possible that we would observe stronger identifiability for lower values (say between 0 and 2), where extra edges are more likely to be involved in the epidemic and have an impact on the transmission tree. One way to achieve this would be to draw *m* separately for each node from a specified distribution.

As noted by Lintusaari et al. (2016), uniform priors on model parameters may translate to highly informative priors on quantities of interest. We observed a non-linear relationship between the PA power *α* and the power law exponent *γ* (Supplementary Fig. S15). Therefore, placing a uniform prior on *α* between 0 and 2 is equivalent to placing an informative prior that *γ* is close to 2. Therefore, if we were primarily interested in *γ* rather than *α*, a more sensible choice of prior might have a shape informed by Supplementary Figure S15 and be bounded above by ∼*α* = 1.5. This would uniformly bound *γ* in the region 2≤γ≤4 commonly reported in the network literature (Colgate et al. 1989; Liljeros et al. 2001; Schneeberger et al. 2004; Leigh Brown et al. 2011). We note however that Jones and Handcock (Jones and Handcock 2003) estimated *γ* values greater than four for some datasets, in one case as high as 17, indicating that a wider range of permitted *γ* values may be warranted.

### Analysis of Real World HIV Datasets

Our investigation of published HIV datasets indicated heterogeneity in the contact network structures underlying several distinct local epidemics. When interpreting these results, we caution that the BA model is quite simple and most likely misspecified for these data. In particular, the average degree of a node in the network is equal to 2*m*, and therefore is constrained to be a multiple of 2. Furthermore, we considered the case *m* = 1, where the network has no cycles, to be implausible and therefore assigned it zero prior probability in one set of analyses. This forced the average degree to be at least four, which may be unrealistically high for sexual networks. The fact that the estimated values of *α* differed substantially for several datasets depending on whether or not *m* = 1 was allowed by the prior is further evidence of this potential misspecification. However, we note that the ordering of the datasets with respect to *α* was similar between the two priors, and the estimates of *I* were robust to the choice of prior for all datasets studied (compare Fig. 5 and Supplementary Fig. S11). More sophisticated models, for example models incorporating heterogeneity in node behaviour, are likely to provide a better fit to these data.

### PA Power Is Sub-Linear and Higher for IDU Networks

For all datasets we examined, the posterior mean estimates for *α* were sub-linear, ranging from 0.27 to 0.83. The sub-linearity is consistent with the results of de Blasio, Svensson, and Liljeros (2007), who developed a statistical inference method to estimate the parameters of a more sophisticated PA model incorporating heterogeneous node behaviour. When used to analyze population-level longitudinal partner count data, they found *α* values ranging from 0.26 to 0.62 depending on the gender and time period considered. Based on these findings, de Blasio, Svensson, and Liljeros (2007) asserted that PA may not fully explain the scale-free networks observed in real life. Other mechanisms, such as homophily, may play a role in generating these networks. Further epidemiological investigation is needed in order to to gain a more thorough understanding of the behaviours that shape contact networks in human populations.

Both de Blasio, Svensson, and Liljeros (2007) and the HET/Botswana data studied populations whose primary risk factor for HIV infection was heterosexual contact. de Blasio, Svensson, and Liljeros (2007) explicitly excluded reported homosexual contacts; Novitsky et al. (2014) did not, but noted that heterosexual contact is the primary mode of transmission in Botswana where the study was done. In the first of the two papers describing the Botswana study (Novitsky et al. 2013), the authors noted that their sample was gender-biased, being composed of ∼75% women. Our estimate of *α* for these data was 0.55 or 0.53, depending on the prior on *m*. Similarly, de Blasio, Svensson, and Liljeros (2007) estimated 0.54, 0.57, and 0.29 for 3-, 5-year, and lifetime partnership networks respectively for the female portion of their sample.

The datasets derived from IDU populations had a higher estimated PA power than the other datasets (Fig. 5 and Supplementary Fig. S11). This finding is in line with Dombrowski et al. (2013), who reanalyzed a network of IDUs in Brooklyn, USA, collected between 1991 and 1993 (Friedman. et al. 2006). They found that the IDU network resembled a BA network much more closely than other social and sexual networks, and offered sociological explanations for the apparent PA dynamics in this population. Importantly, from a public health perspective, the authors asserted that the removal of *random* individuals from IDU networks may have the paradoxical effect of increasing the networkin epidemic susceptibility. When low-degree nodes are removed, as would occur during a police crackdown, their network neighbours may turn to well-known community members for advice or supplies, thus increasing the connectivity of these high-degree nodes.

Unfortunately, the sub-linear region for *α* identified by both de Blasio, Svensson, and Liljeros (2007) and *netabc* is also the region of poorest identifiability (Fig. 2). This was reflected in the high level of uncertainty in the estimates, with most 95% HPD intervals covering the majority of the range [0, 1]. The value α=0.5 was contained in the 95% HPD interval for every dataset; consequently, it is not possible to say with high confidence that any of the *α* values are different from each other. In synthetic data, the confidence intervals around *α* narrowed when other parameters were marginalized out (Supplementary Fig. S9). Thus, it is possible that estimates of *α* could be made more precise by specifying either exact values or informative priors on the other BA parameters when these are known.

### Other BA Parameters

The true HIV prevalence in a population can be difficult to estimate for several reasons. HIV-infected individuals may be asymptomatic for months or years, possibly delaying their awareness of their status. In many contexts, the risk factors for acquisition of HIV are illegal or stigmatized, which may represent a barrier to testing, treatment, and/or disclosure of status. Our simulation study showed that *I* is weakly identifiable from tree shapes, however, the estimates of *I* obtained with *netabc* were upwardly biased (Fig. 2). In addition, our initial exploratory analysis showed that the identifiability of *I* decreases with the number of sampled tips (Supplementary Fig. S6); in real world studies, the proportion of infected individuals sampled is usually low. The estimated *I* values for the HIV datasets ranged from 373 (IDU/Estonia) to 5,432 (HET/Botswana). For the IDU/Estonia data, considering duplicated sequences which were initially removed caused the estimate of *I* to increase nearly twofold. If these duplicated sequences truly represent distinct patients, the estimate of *I* when they are included is likely more accurate.

We were not able to discern and trends toward over- or underestimation of *I* from available prevalence data. For example, the authors of the HET/Botswana data (Novitsky et al. 2013, 2014) estimated that there were 1,731 HIV-positive individuals in the study area. HIV sequences were obtained from ∼70% of these individuals. The estimated prevalence we obtained was much higher (5432), with a 95% HPD interval spanning nearly the entire prior region. On the other hand, the study which produced the MSM/USA data (Little et al. 2014) enrolled 648 HIV-positive MSM; thus, our estimate of *I* (482) was clearly an underestimate. *Post-hoc* explanations can be imagined for both of these results. The HET/Botswana data were collected from a town located proximally to the country's capital city; the authors suggested that frequent travel between the two locations may have facilitated linking of their sexual networks. Thus, the high estimate of *I* we obtained for these data may include a larger network component based in the capital city. The MSM/USA result may indicate that the individuals genotyped for the study are members of a smaller subnetwork which does not include the entire local MSM population. Unfortunately, none of these hypotheses can be easily tested.

Over half of the datasets were estimated to have *m* = 1, which produces tree-like networks without cycles. Since the average degree of a BA network is 2*m*, this value may simply reflect the fact that most people have a small number of sexual partners, especially when only recent partnerships are considered (Liljeros et al. 2001). In fact, in one survey, the most common number of partnerships in the past 12 months was one (Liljeros et al. 2001); the BA model does not allow any nodes with degree 1 when m≥2. For this reason, the choice of whether to allow m=1 in the prior is problematic, as we must choose between an unrealistic topology (no cycles) and an unrealistic minimum degree. Extensions to the BA model which relax this constraint can be imagined and may offer improved parameter resolution. Estimates of *N* were not informative for any of the datasets under either choice of prior, consistent with our simulation results.

### Modelling Assumptions

In addition to the aforementioned possibility of misspecification, additional modelling assumptions include the network being connected and static, all transmission rates being equal, no removal after infection, identical behaviour of all nodes, and random sampling. The last two were addressed with small-scale experiments. We simulated a network where some nodes exhibited a higher attachment power than others, and found that the estimated attachment power was simply the average of the two values. This indicated that, although we could characterize the network in aggregate, the estimated parameters could not be said to apply to any individual node. The effect of biased sampling was investigated by analyzing a transmission tree which had been sampled in a peer-driven fashion. The results were roughly in line with those for random sampling, however the estimated value of *α* was lower than the average for randomly sampled trees. Further experiments would be necessary to fully explore the impact of these assumptions on the methodti accuracy. However, despite these issues, we felt it was best to demonstrate the method first on a simple model. It is possible to use this framework to fit more complex models which address some of these issues, such as one incorporating heterogeneous node behaviour, which may prove a fruitful avenue for future investigations.

Our method has a number of caveats, perhaps the most significant being that it takes a transmission tree as input. In reality, true transmission trees are not available and must be approximated, often by way of a viral phylogeny. Although this has been demonstrated to be a fair approximation [e.g. Leitner et al. 1996], and is frequently used in practice [e.g. Stadler and Bonhoeffer 2013], the topologies of a viral phylogeny and transmission tree can differ significantly (Ypma, Marijn van Ballegooijen, and Wallinga 2013) due to within-host evolution and the sampling process (Giardina 2016). The ABC-SMC algorithm is computationally intensive, taking about a day when run on 20 cores in parallel with the settings we described in the methods. Nevertheless, our method is potentially useful to epidemiological researchers interested in the general characteristics of the network structure underlying disease outbreaks. This work, and previous work by our group (Poon 2015), has demonstrated that ABC is a broadly applicable and effective framework in which to perform phylodynamic inference.

## Supplementary data

Supplementary data are available at *Virus Evolution* online.

Supplementary Fig. S1

## Availability

C source code for *netabc* is available at github.com/rmcclosk/netabc. Scripts to run all computational experiments are available at git.io/vKVsX.
