# Supplementary material for: Reconstructing contact network parameters from viral phylogenies
Source: Virus Evol. 2016 Oct 30;2(2):vew029. doi: 10.1093/ve/vew029 (PMC5094293; doi:10.1093/ve/vew029)
Supplement: Supplementary Fig. S1 [file supp.pdf]

Supplementary Figures

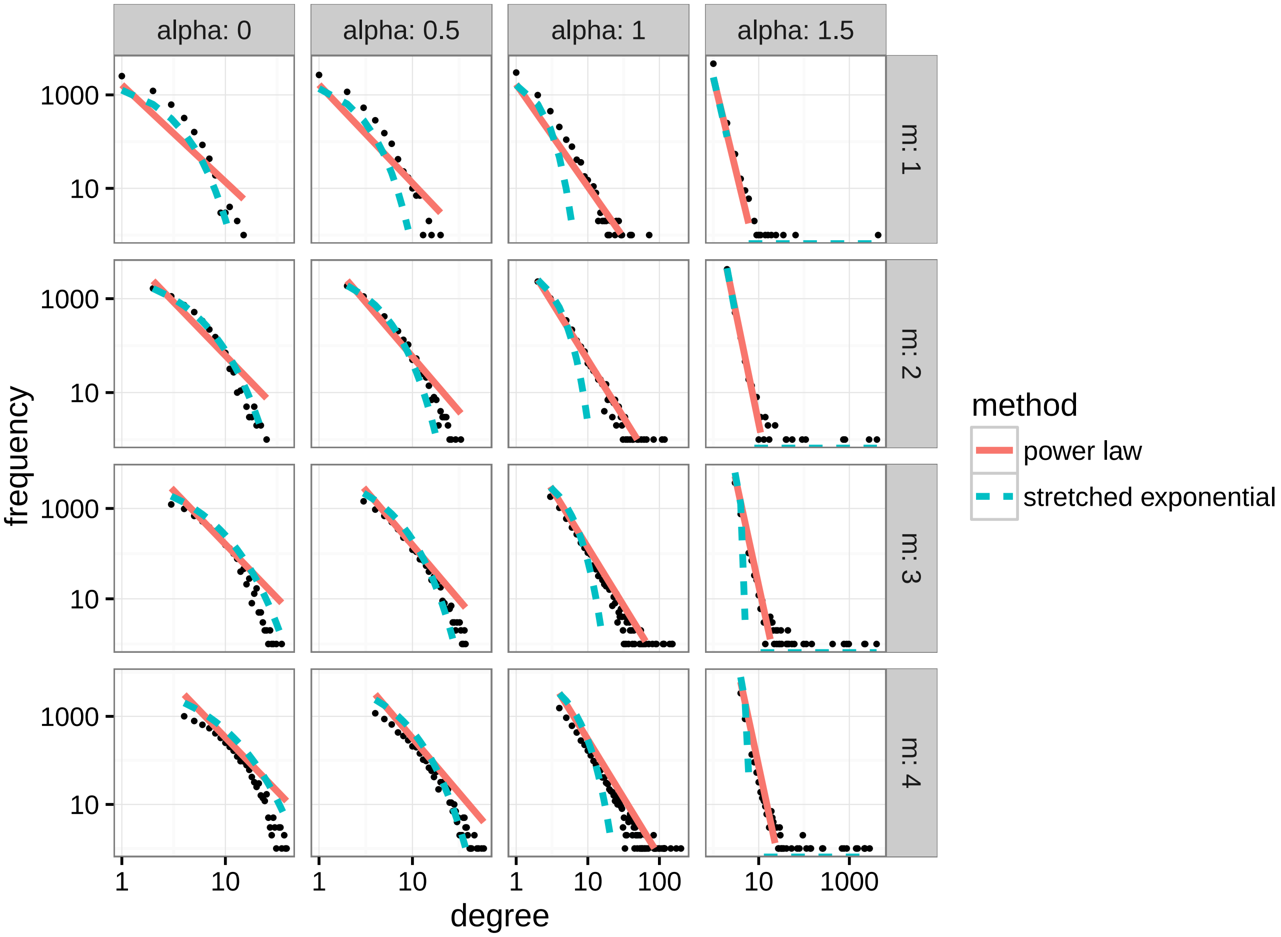

Figure S1: Best fit power law and stretched exponential curves for degree distributions of simulated BA networks. Sixteen networks of size $N = 5000$ were simulated under the BA model with various values of the preferential attachment power α and mean degree $2m$. Empirical degree distributions of each network are indicated by black dots. Solid red lines indicate best-fit power law distributions (true distribution for $\alpha = 1$); dashed blue lines indicate best-fit stretched exponential distributions (true for $\alpha < 1$).

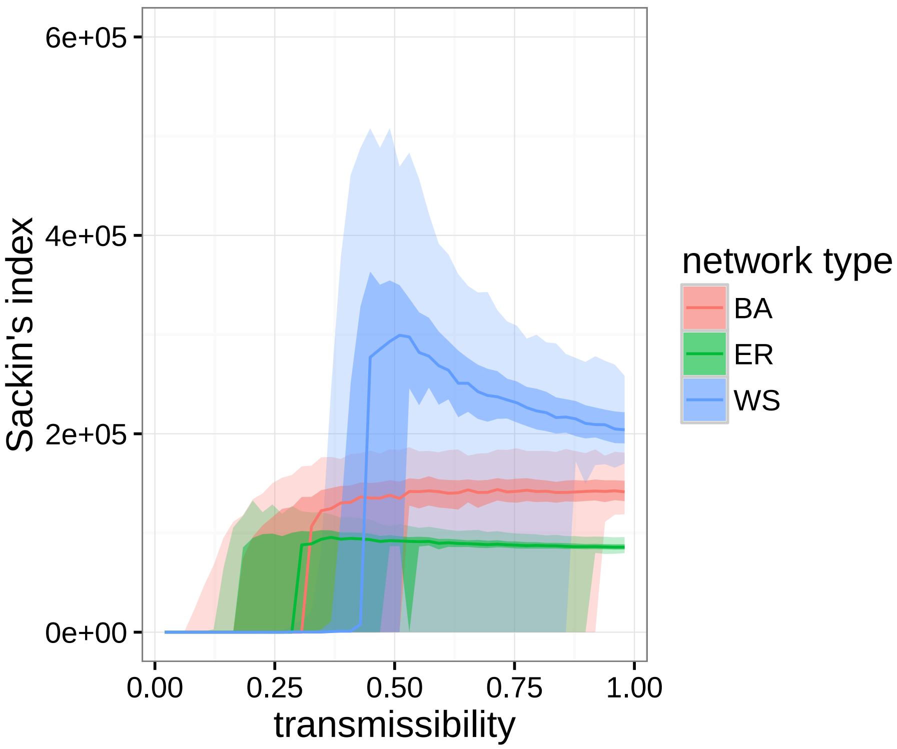

Figure S2: Reproduction of Figure 1A from Leventhal *et al.* (2012) used to check the accuracy of our implementation of Gillespie simulation. Transmission trees were simulated over three types of network, with pathogen transmissibility varying from 0 to 1 (1000 trees per transmissibility value). Sackin's index was calculated for each simulated transmission tree. Lines are means, light shaded areas are 95% quantile range, and dark shaded areas are interquartile ranges.

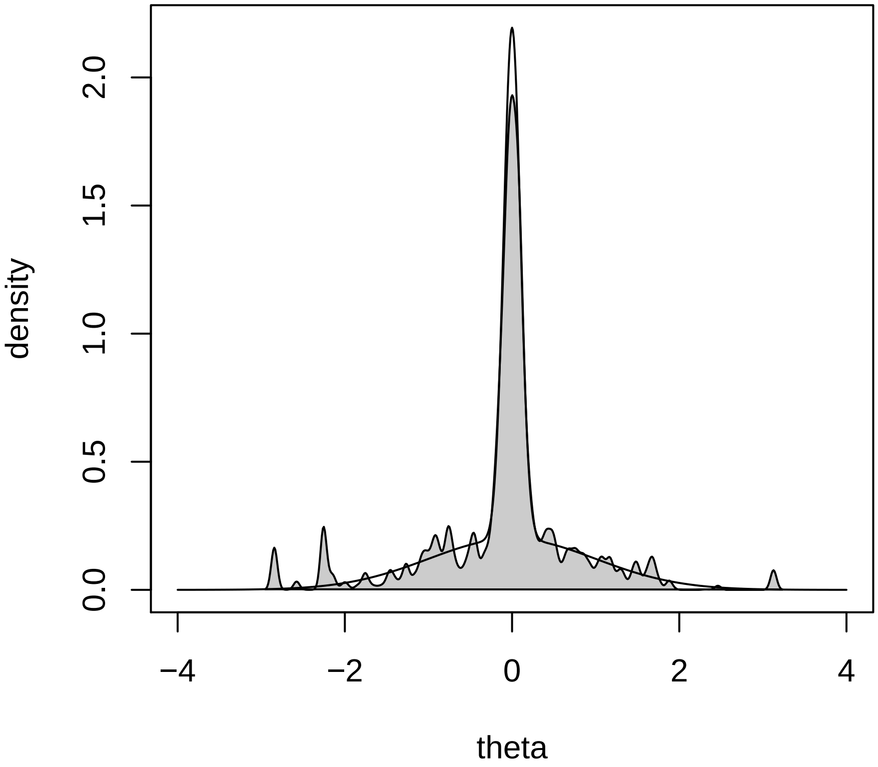

Figure S3: Approximation of mixture of Gaussians used by Del Moral *et al.* (2012) and Sisson *et al.* (2009) to test SMC. Solid black line indicates true distribution. Grey shaded area shows SMC approximation obtained with our implementation, using 10000 particles with one simulated data point per particle.

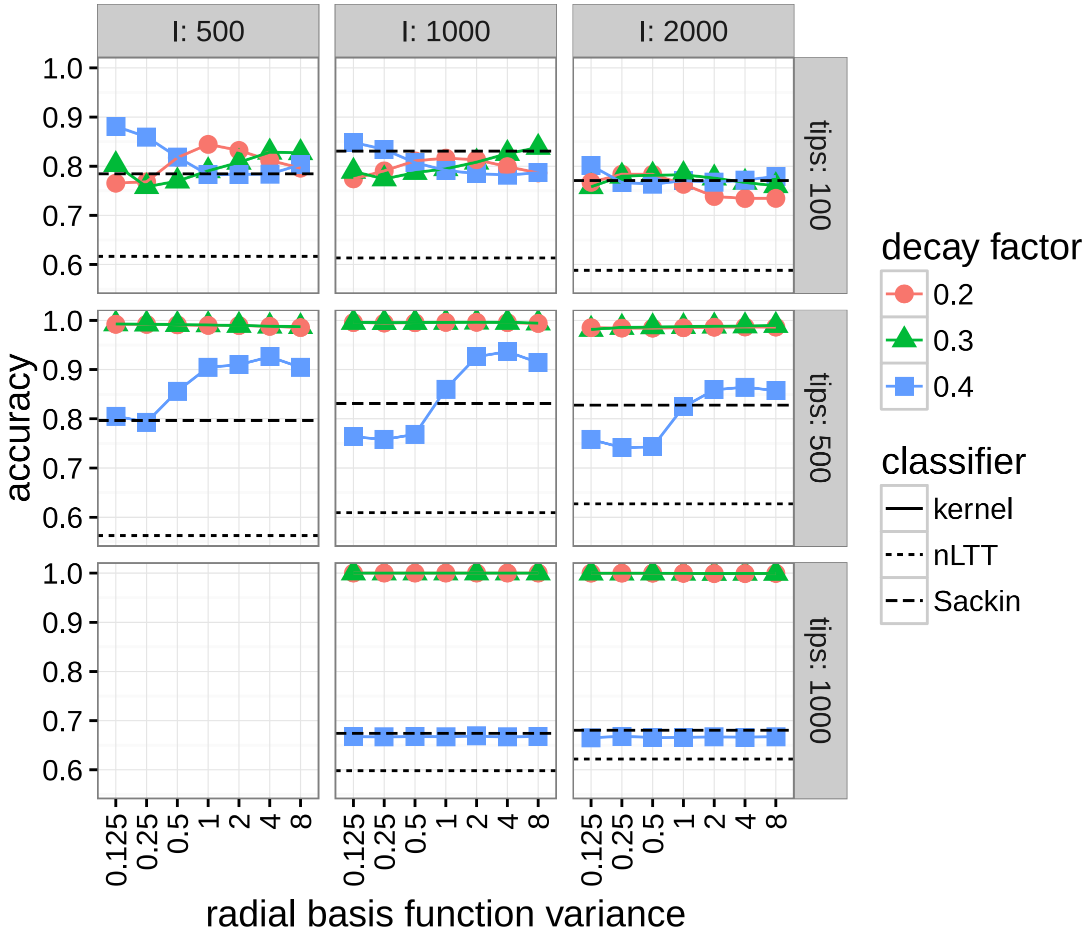

Figure S4: Cross-validation accuracy of kernel-SVM classifiers for α parameter of BA network model, for various tree kernel meta-parameters and epidemic scenarios. Each point was calculated based on 300 simulated transmission trees over networks with $\alpha = 0.5$, 1.0, or 1.5. Dotted and and dashed lines indicate, respectively, performance of SVMs using the nLTT statistic and Sackin's index. Facets are number of infected nodes before the simulation was stopped (I) and number of tips in the sampled transmission tree.

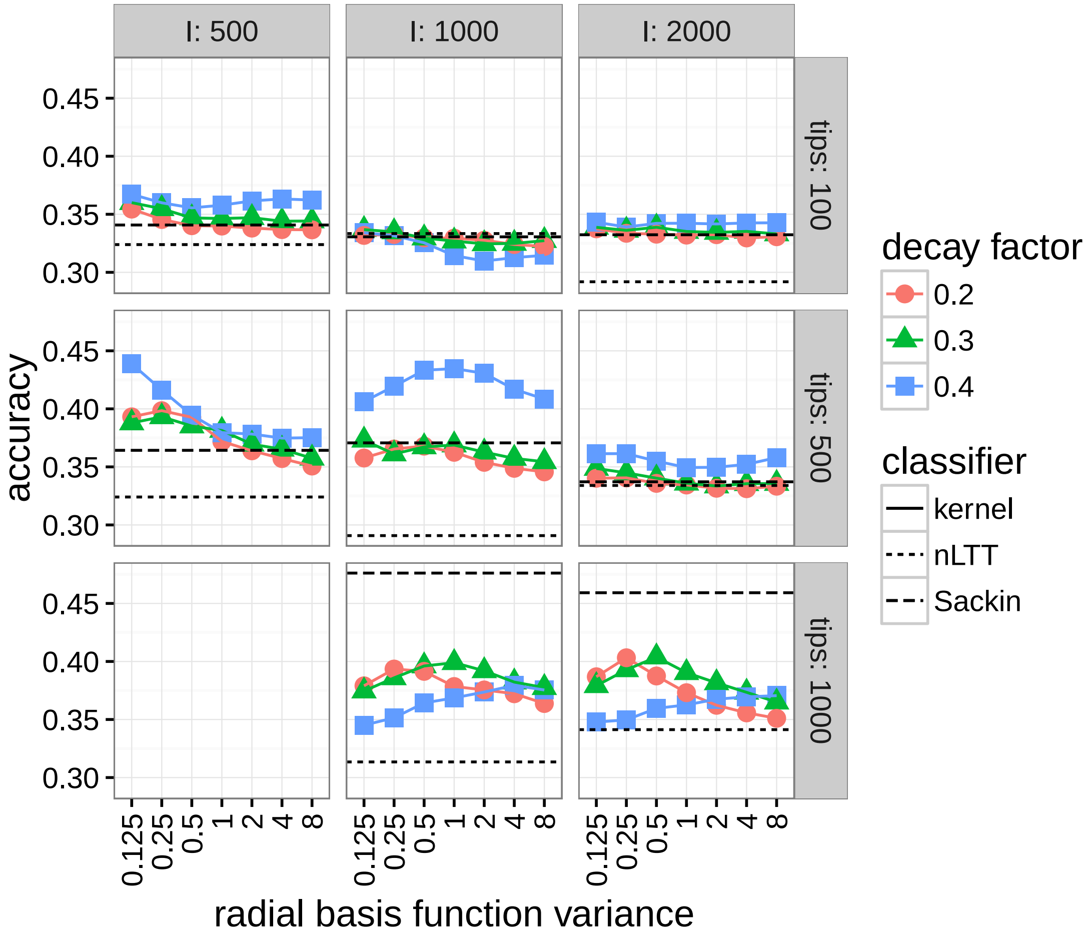

Figure S5: Cross-validation accuracy of kernel-SVM classifiers for m parameter of BA network model, for various tree kernel meta-parameters and epidemic scenarios. Each point was calculated based on 300 simulated transmission trees over networks with m = 2, 3, or 4. Dotted and and dashed lines indicate, respectively, performance of SVMs using the nLTT statistic and Sackin's index. Facets are number of infected nodes before the simulation was stopped (I) and number of tips in the sampled transmission tree.

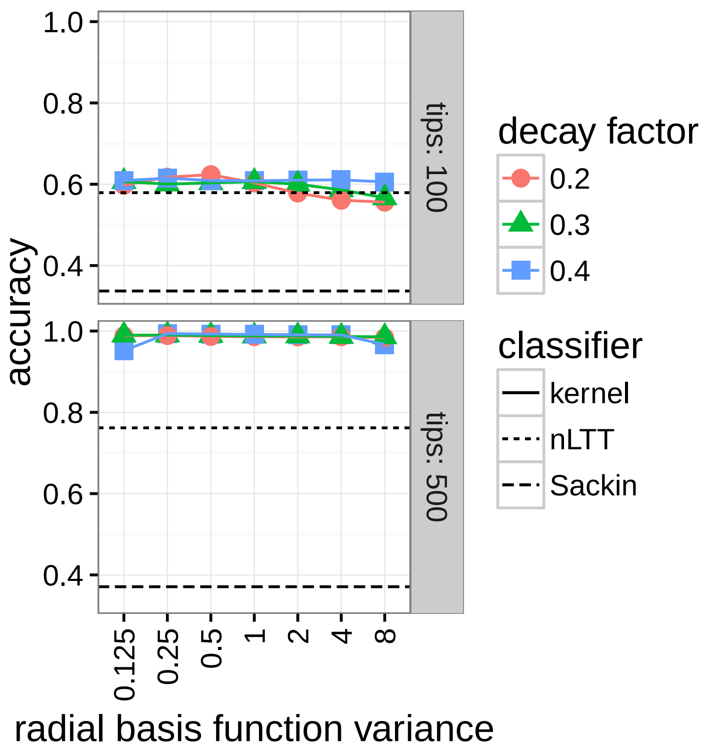

Figure S6: Cross-validation accuracy of kernel-SVM classifiers for number of infected nodes (I) under BA network model, for various tree kernel meta-parameters and two tree sizes. Each point was calculated based on 300 simulated transmission trees over networks with I = 500, 1000, or 2000. Dotted and and dashed lines indicate, respectively, performance of SVMs using the nLTT statistic and Sackin's index. Facets are the number of tips in the sampled transmission tree.

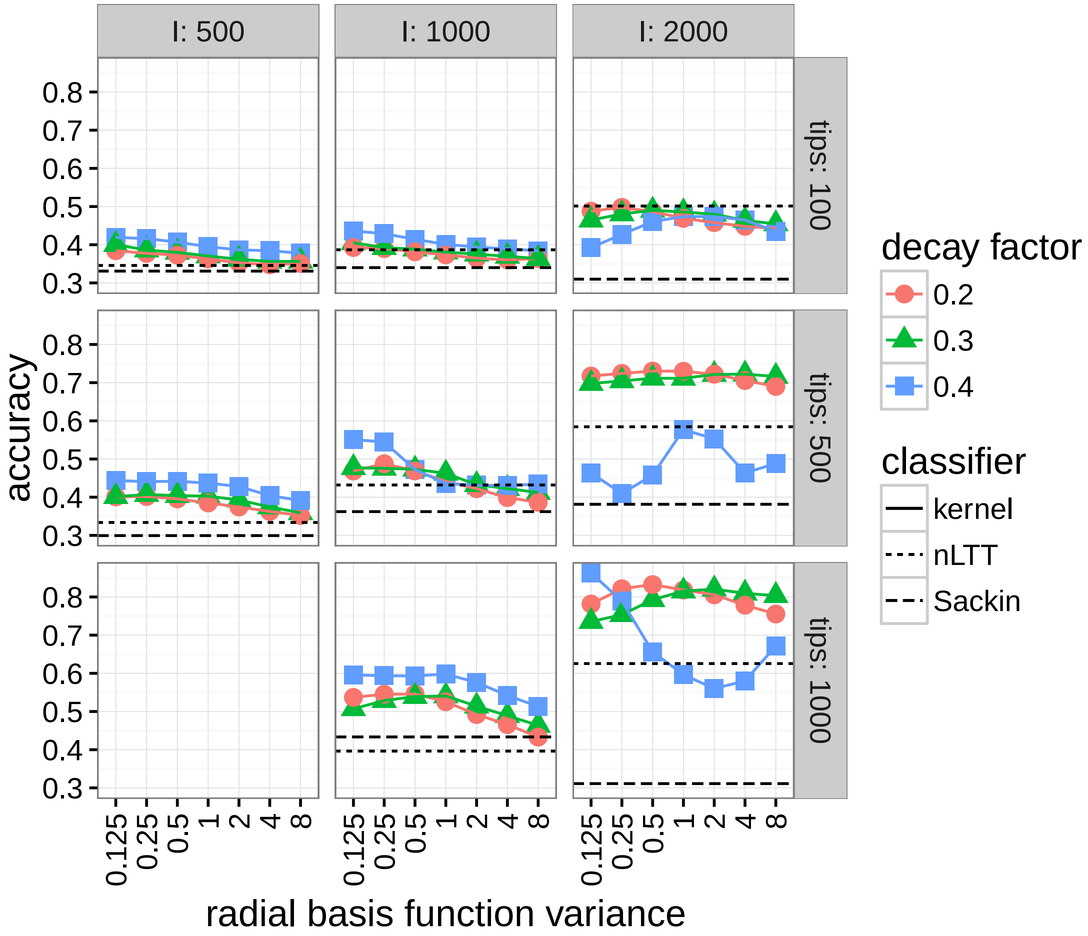

Figure S7: Cross-validation accuracy of kernel-SVM classifiers for total number of nodes (N) under BA network model, for various tree kernel meta-parameters and epidemic scenarios sizes. Each point was calculated based on 300 simulated transmission trees over networks with $N = 3000$, 5000, or 8000. Dotted and and dashed lines indicate, respectively, performance of SVMs using the nLTT statistic and Sackin's index. Facets are the number of tips in the sampled transmission tree.

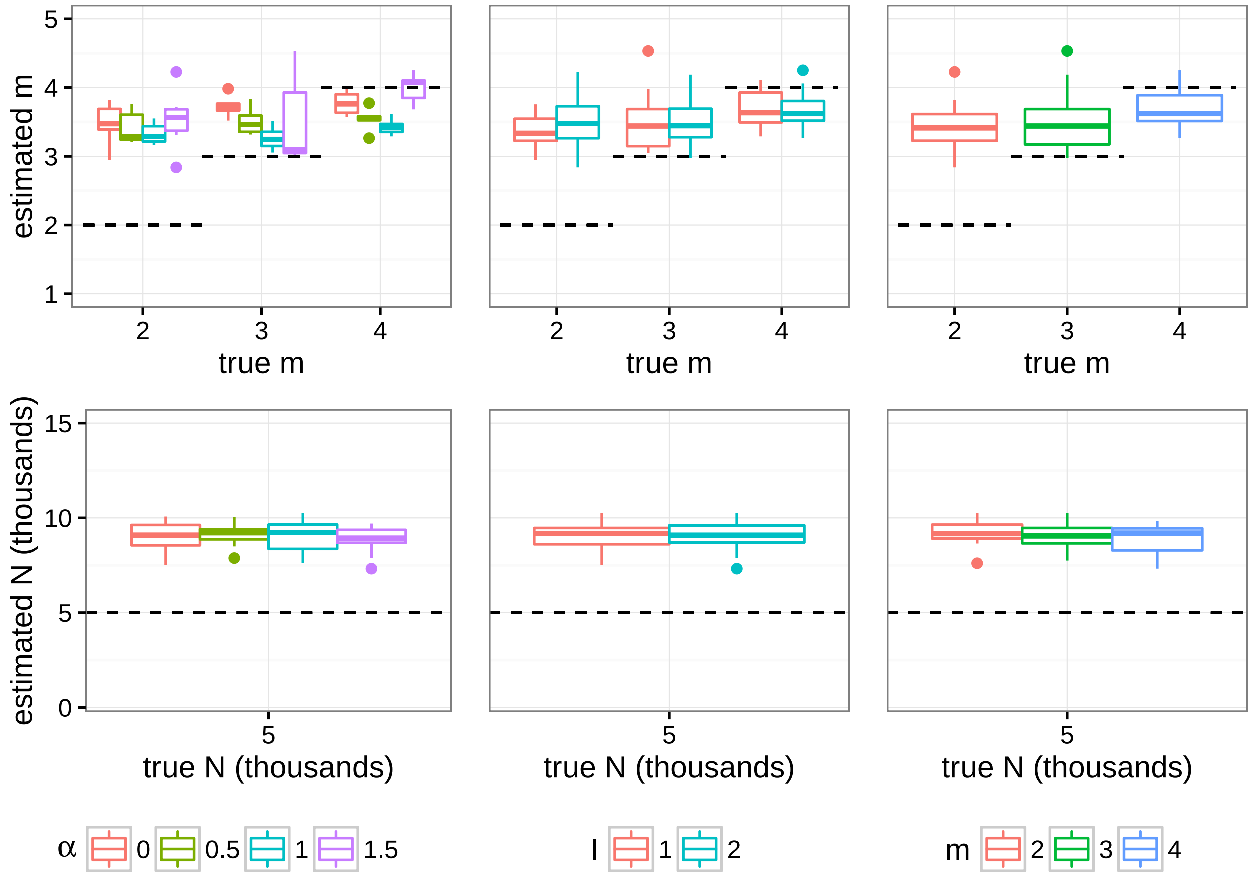

Figure S8: Posterior mean point estimates for BA model parameters m and N obtained by running *netabc* on simulated data, stratified by true parameter values. First row of plots contains true versus estimated values of m; second row contains true versus estimated values of N. Columns are stratified by α, I, and m respectively. Dashed lines indicate true values.

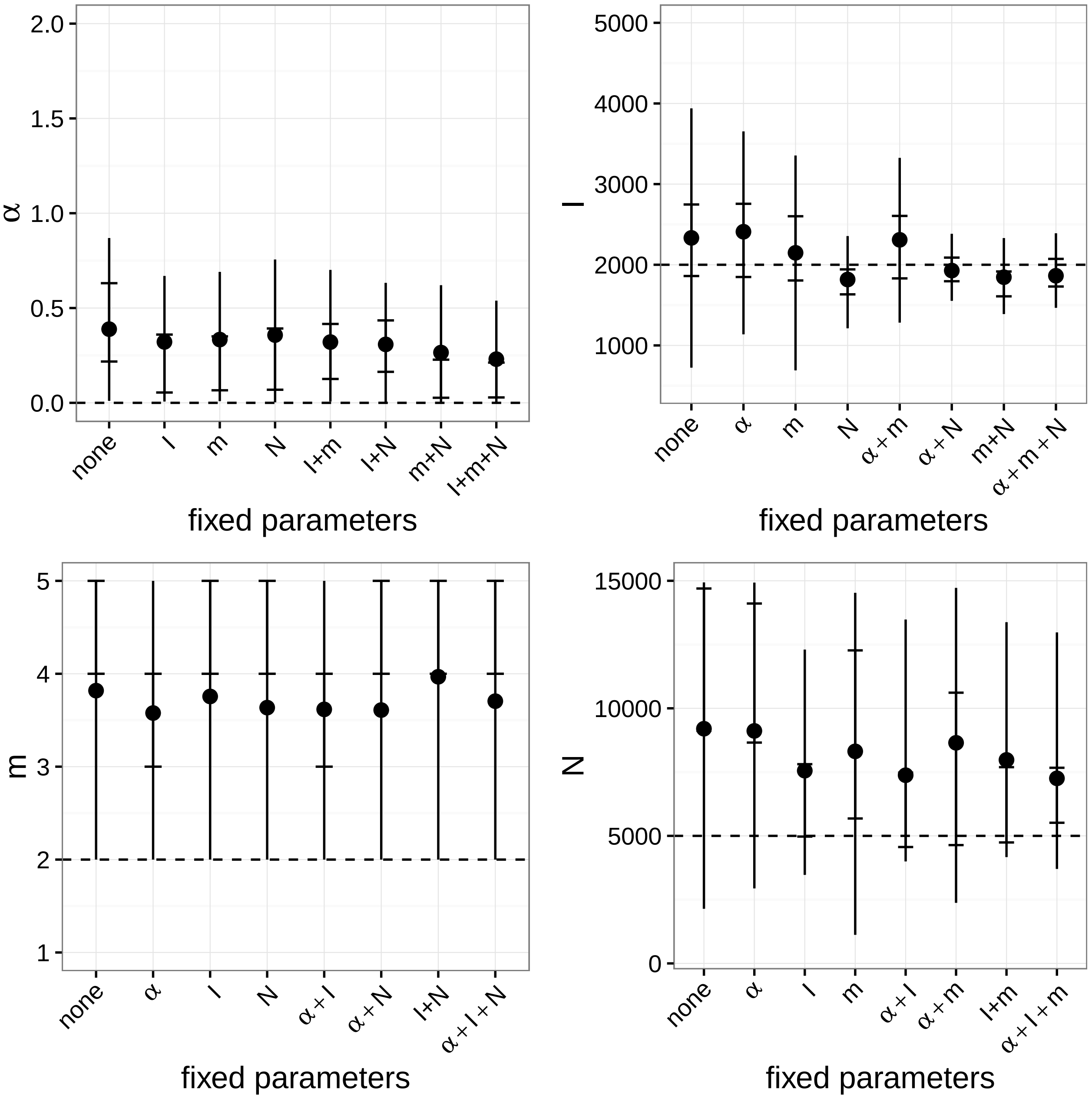

Figure S9: Posterior means (points), 50% HPD intervals (notches), and 95% HPD intervals (lines) for BA model parameters estimated marginally with ABC. *x*-axis labels indicate parameters which were fixed to their true values by specifying Dirac-delta priors.

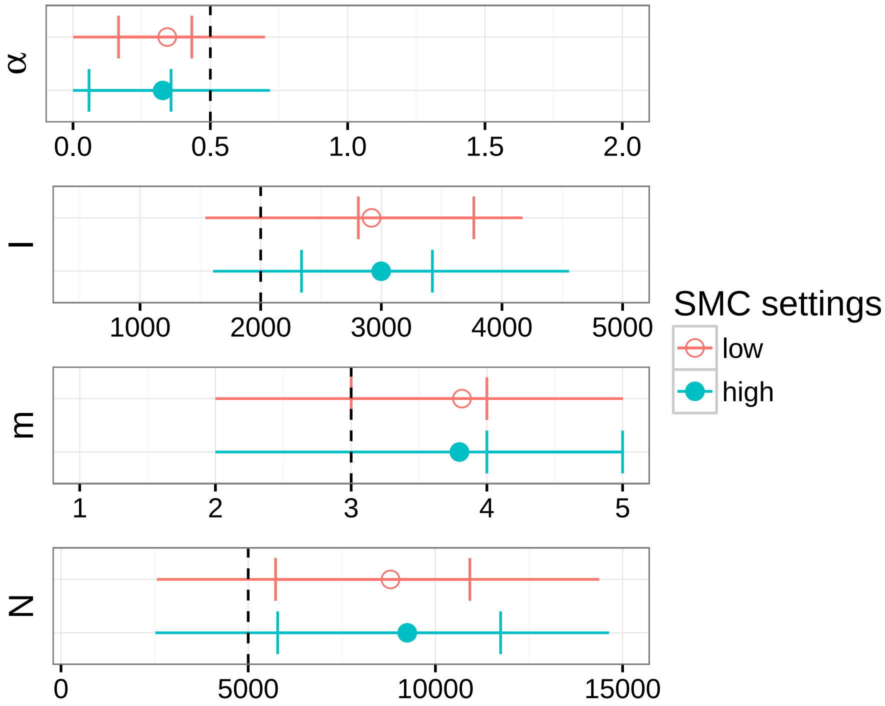

Figure S10: Posterior means (points), 50% HPD intervals (notches), and 95% HPD intervals (lines) for BA model parameter estimated with ABC using two sets of SMC settings. "Low" settings are 1000 particles, 5 simulated datasets per particle, and $\alpha_{\text{ESS}} = 0.95$. "High" settings are 2000 particles, 10 simulated datasets per particle, and $\alpha_{\text{ESS}} = 0.97$.

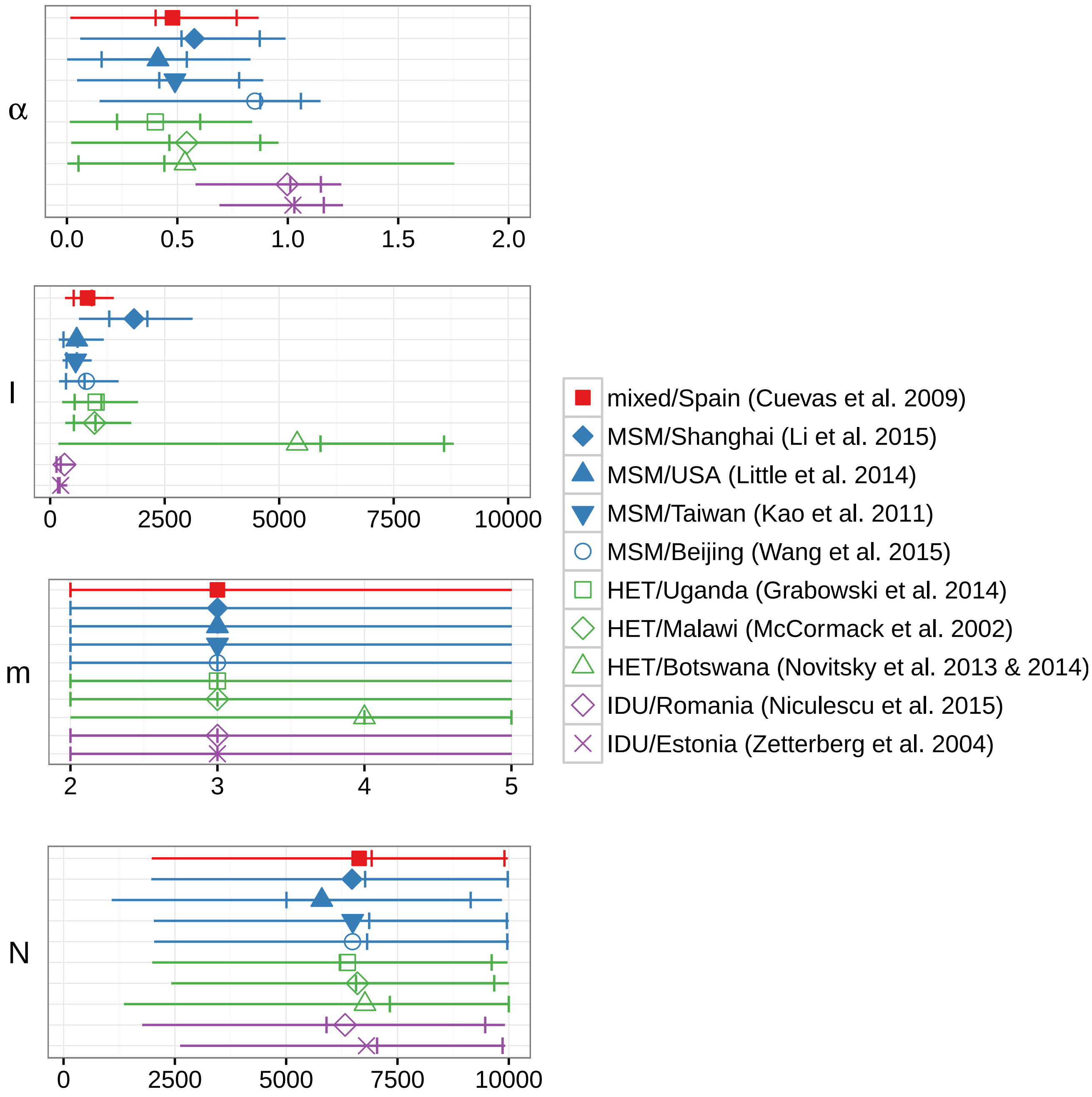

Figure S11: Maximum *a posteriori* point estimates and 95% HPD intervals for parameters of the BA network model, fitted to five published HIV datasets with ABC. x-axes indicate regions of nonzero prior density. In particular, the prior on m was DiscreteUniform(2, 5).

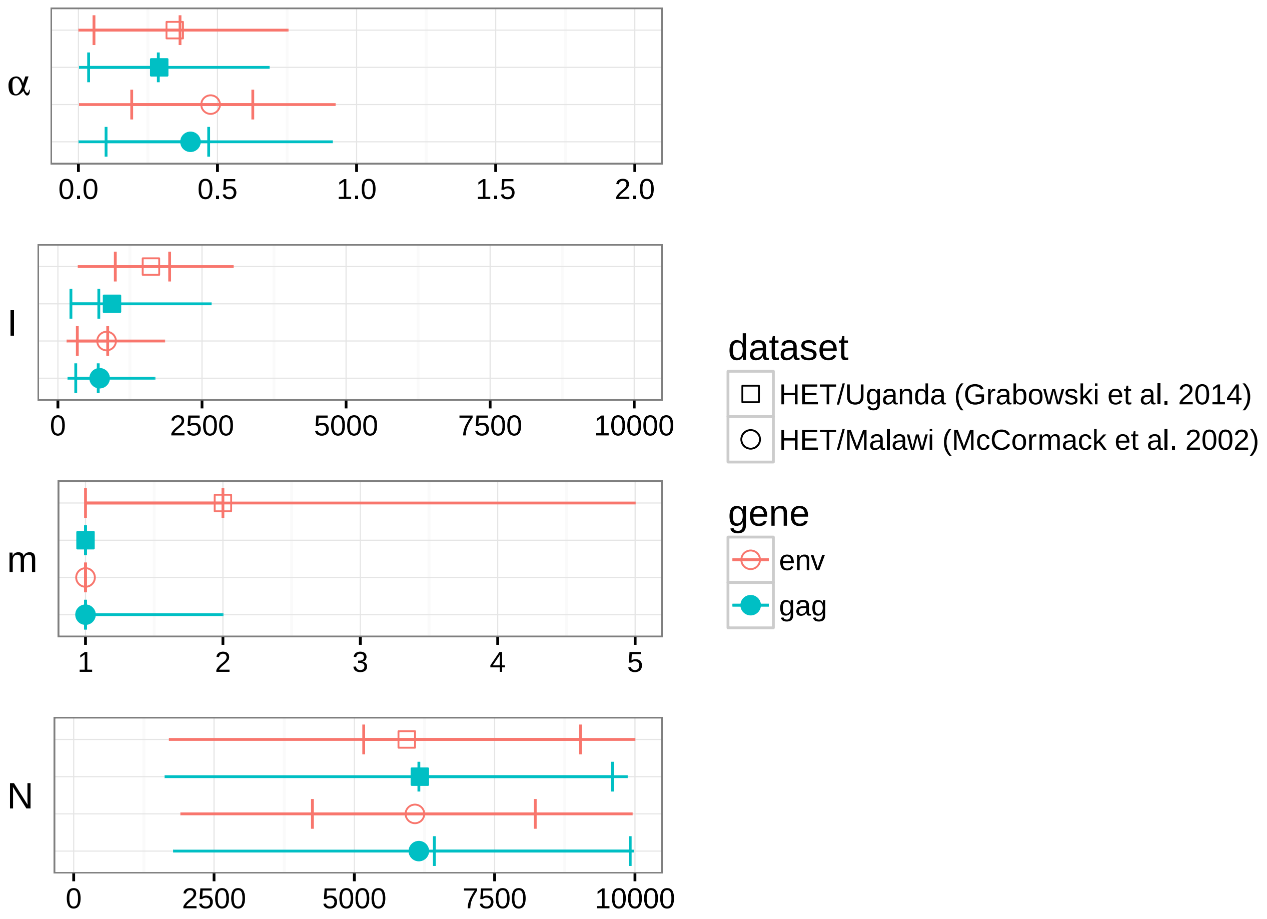

Figure S12: Posterior means (points), 50% HPD intervals (notches), and 95% HPD intervals (lines) for parameters of the BA network model, fitted to two HIV datasets where both *gag* and *env* genes were sequenced.

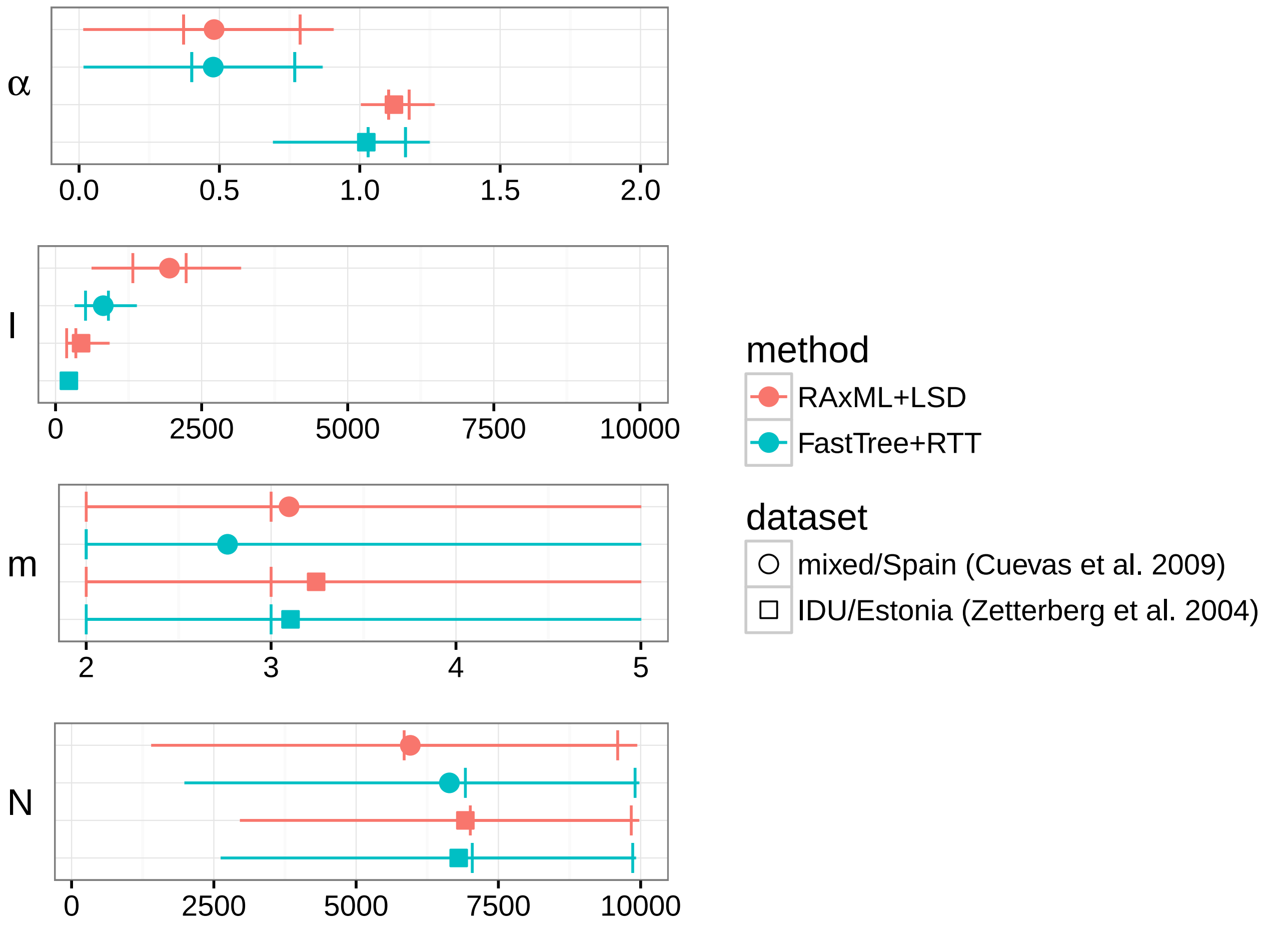

Figure S13: Posterior means (points), 50% HPD intervals (notches), and 95% HPD intervals (lines) for parameters of the BA network model, fitted to two HIV datasets using two phylogenetic reconstruction methods.

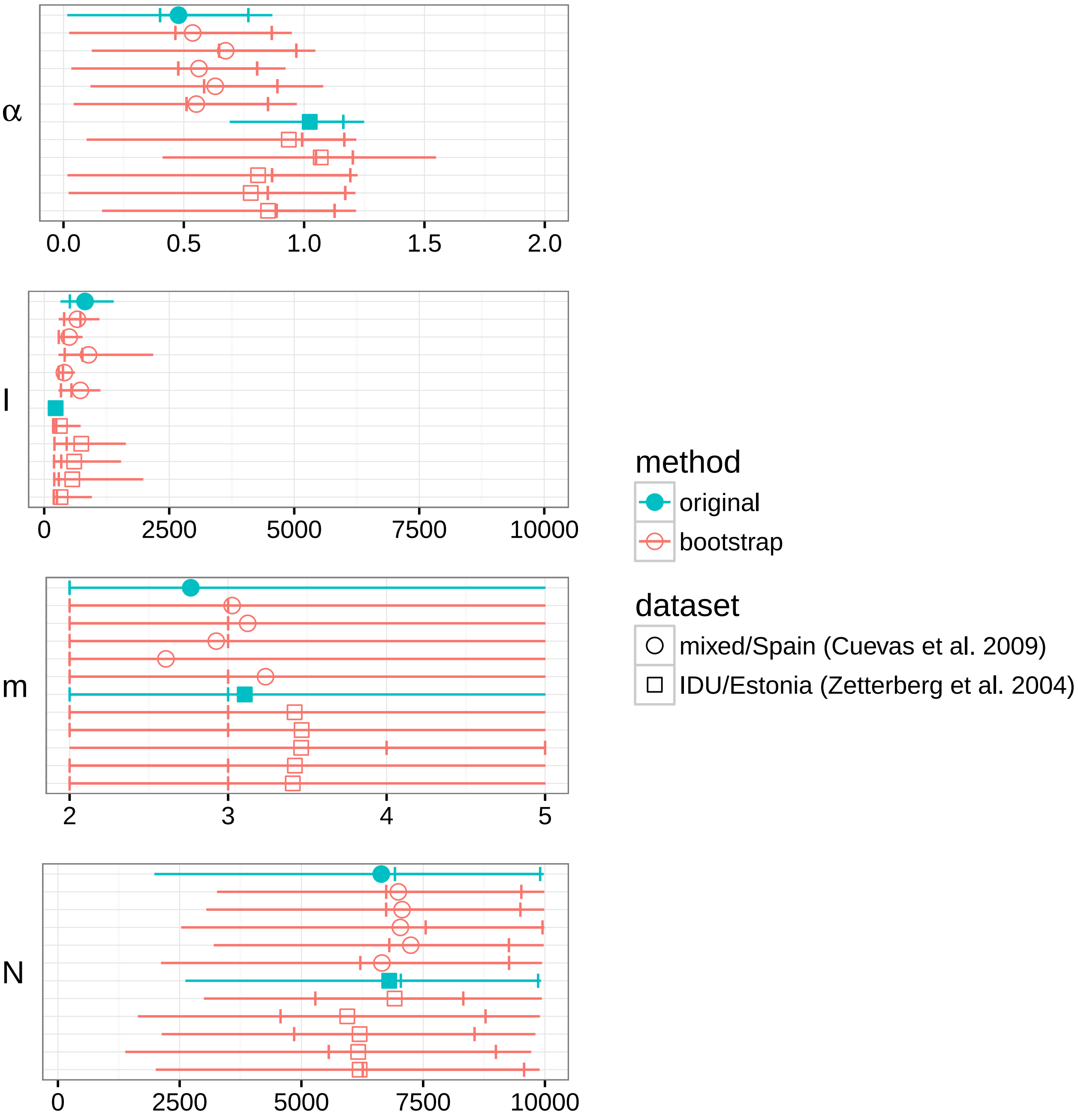

Figure S14: Posterior means (points), 50% HPD intervals (notches), and 95% HPD intervals (lines) for parameters of the BA network model, fitted to original and bootstrap replicate alignments of two HIV datasets.

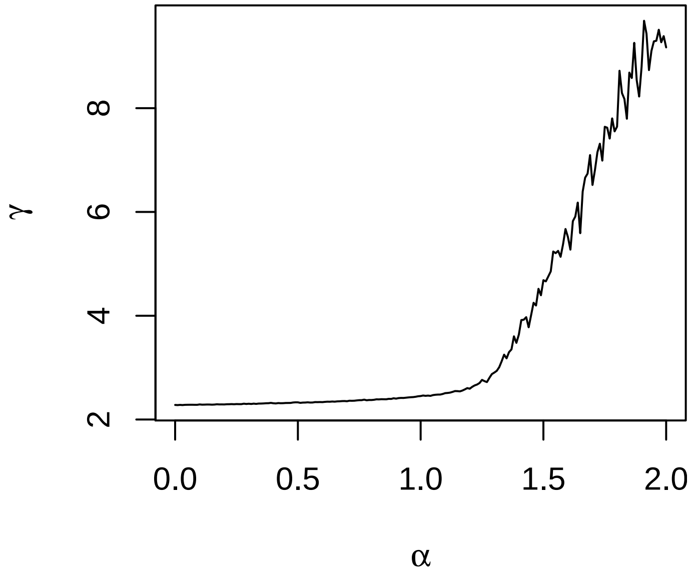

Figure S15: Relationship between preferential attachment power parameter α and fitted power law exponent γ for networks simulated under the BA network model with $N = 5000$ and $m = 2$. Note that the degree distribution truly follows a power law only when $\alpha = 1$ (see fig. S1).

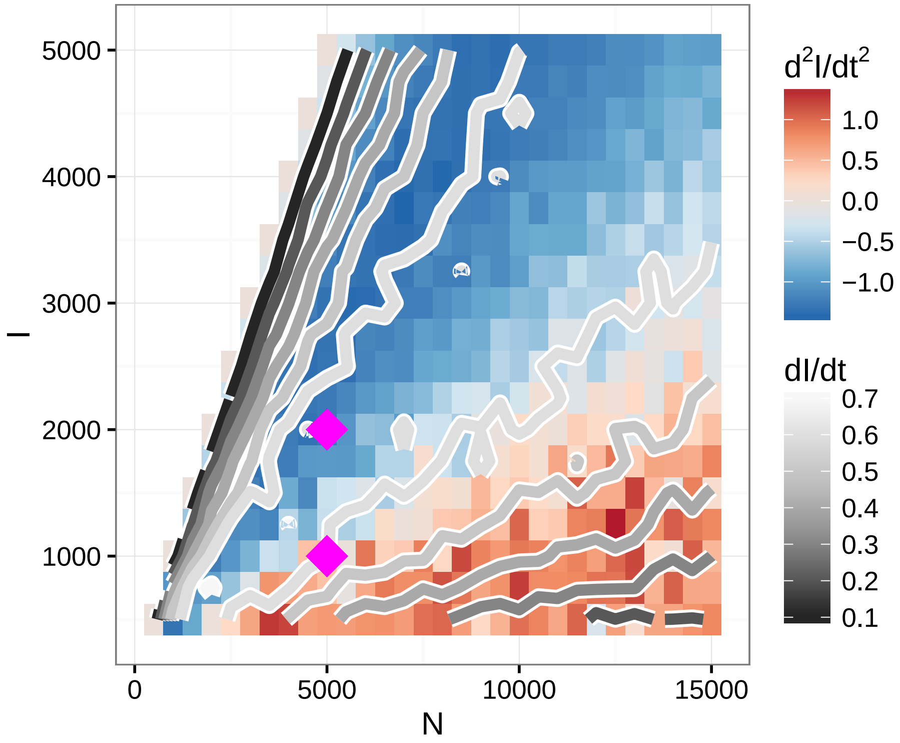

Figure S16: First and second time derivatives of epidemic growth curves at time of sampling for various values of I and I. Networks were simulated under the BA model with $\alpha = 1.0$, $m = 2$, and N varied along the values shown on the x-axis. Transmission trees were sampled at the time when I nodes were infected (y-axis). Logistic growth curves were fit to epidemic trajectories derived from the transmission trees, and their first and second derivatives were calculated at the time of sampling. Contours show first derivatives, while colours indicate second derivatives. Values of I and N used in simulation experiments with ABC are indicated by diamonds.

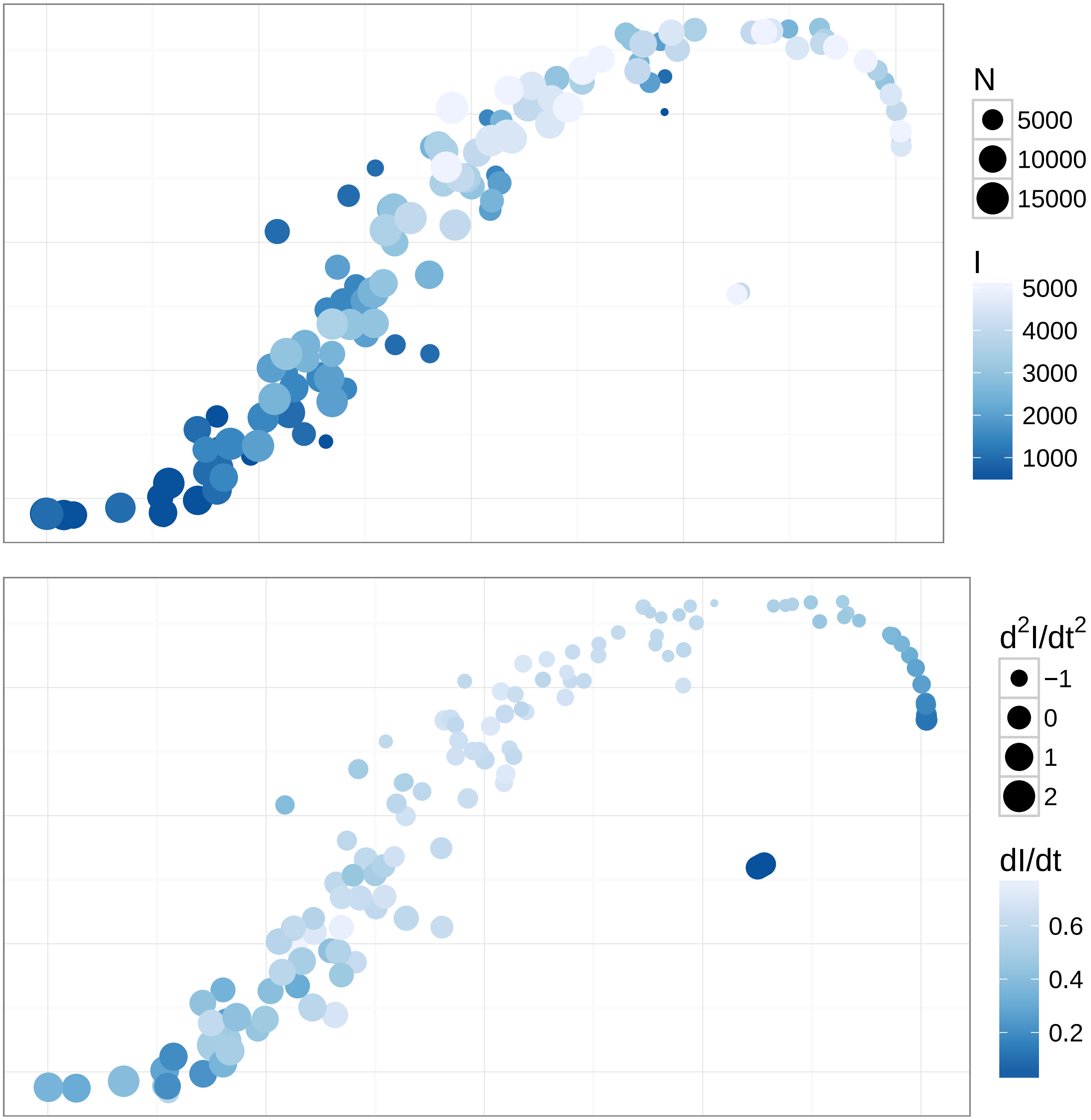

Figure S17: Two-dimensional embedding of transmission trees simulated over BA networks for various values of the parameters I and N. The embedding was created with t-distributed stochastic neighbor embedding (t-SNE) using the tree kernel as a distance function. Sizes and colours of points indicate (top) values of I and N used to simulate the trees, or (bottom) first and second time derivatives of epidemic growth curves corresponding to simulated trees. Trees with similar shapes, whose corresponding points are close together in the embedding, tend to share similar first and second derivatives.

1 Supplementary Text S1

We present here a non-rigorous justification for the use of ABC for the problem at hand, as opposed to more frequently-used approaches for fitting mathematical models. Consider a contact network model with parameters θ, and an estimated transmission tree T. Taking a Bayesian approach, our aim is to obtain a sample from the posterior distribution on the model's parameters given our data,

$$\pi(\theta \mid T) = \frac{f(T \mid \theta)\pi(\theta)}{\int_{\Theta} f(T \mid \theta)\pi(\theta) d\theta}.$$

For all but the simplest models, the normalizing constant in the denominator is an intractable integral. What we shall argue here is that, in contrast to most commonly studied mathematical models, the likelihood $f(T \mid \theta)$ is also likely to be intractable in our case.

The internal nodes of transmission trees represent transmission events, and are labelled with the donor in the associated transmission pair. However, when we estimate a transmission tree from viral sequence data, we generally only know the labels of the tips of the tree, not the labels of the internal nodes. In viral phylogenies, the transmissions are at least partially preserved through the evolutionary relationships among the viruses, but the directionality of those transmissions is unknown. Thus, a single estimated transmission tree can correspond to many possible pathways of the epidemic through the network. When calculating a likelihood given a transmission tree, we must sum over all possible labellings of the internal nodes. Let Ł be the set of such labellings. Then

$$f(T \mid \theta) = \sum_{l \in Ł} f(T, l \mid \theta). \tag{1}$$

A contact network model assigns a probability density to each possible contact network. Transmission trees are realized over particular contact networks, not over the model itself. Therefore, we must also sum over all contact networks which could be generated by the model. Let $\mathscr{G}$ be the set of all possible contact networks. Summing **??** over $\mathscr{G}$ gives

$$f(T \mid \theta) = \sum_{G \in \mathscr{G}} \sum_{l \in Ł} f(T, l \mid G, \theta) f(G \mid \theta). \tag{2}$$

This can be simplified somewhat by noticing that, given a specific contact network, the labelled transmission tree depends only on that network and not on the model that generated it. That is, $f(T, \nu \mid G, \theta) = f(T, l \mid G)$, and

$$f(T \mid \theta) = \sum_{G \in \mathscr{G}} f(G \mid \theta) \sum_{l \in Ł} f(T, \nu \mid G) \tag{3}$$

Under the assumption that both transmission and removal are Poisson processes, calculating

$f(T, l \mid G)$ can be accomplished by a straightforward modification of the Gillespie simulation algorithm. Rather than choosing transmission or removal events according to their probabilities, the events would be deterministically chosen to mimic the events recorded in the transmission tree. Assuming efficient data structures for storing lists of nodes and edges, the complexity of this calculation would be $O(|T|)$. The number of possible labellings of internal nodes of T is easily seen to be $2^{(|T|-1)/2}$ by noticing that each of the $(|T|-1)/2$ internal nodes must be labelled with the same label as either its right child or its left child. Although exponential calculations of this nature can often be simplified on trees using dynamic programming, it cannot be straightforwardly applied in this case because the subtrees' probabilities depend on the existing epidemic progress (their parents and siblings). Hence, calculating the inner sum over labels may take time $O(2^{(|T|-1)/2})$.

The outer sum, over all contact networks, is also difficult to evaluate in general. There are $2^{N(N-1)}$ directed graphs on N nodes. There must be at least as many nodes in the contact network as the number of tips in the tree, which is $(|T|+1)/2$. Of course, it is very likely that there are more nodes in the network than observed tips because some individuals are never infected and/or some infected individuals are never sampled. The complexity of calculating $f(G \mid \theta)$ is obviously dependent on the particular model being investigated. For the BA model, we might have to sum over all possible orders in which the nodes could be added, and all assignments of edges to the nodes which generated them. However, even in the case that calculating $f(G \mid \theta)$ can be done in constant time, the sum (3) still has at least $O(2^{|T|^2})$ terms.

We have shown that both the normalizing constant $\int_\Theta f(T \mid \theta)\pi(\theta)d\theta$ and the likelihood $f(T \mid \theta)$ are likely computationally prohibitive to calculate. If this is the case, the problem of fitting contact network models to phylogenies seems to be of the *doubly-intractable* type, which would imply that these models are amenable to neither ML nor Bayesian inference techniques. Although both methods are able to cope with an intractable normalizing constant (for example, by local search for ML or Bayesian MCMC), neither can avoid the intractable likelihood calculations. This justifies the use of ABC, which is a likelihood-free method.
